# Supplementary material for: Genome-Wide Identification and Evolution Analysis of the Gibberellin Oxidase Gene Family in Six Gramineae Crops
Source: Genes (Basel). 2022 May 12;13(5):863. doi: 10.3390/genes13050863 (PMC9141362; doi:10.3390/genes13050863)
Supplement: Supplementary file 1 [file genes-13-00863-s001.zip › Supplementary file 1.pdf]

>KQJ85786
LPTLDLEEGLTEALAAACRDLGVFRLAN--HGIPAD--LSDRLFSLTRHLL-EEPDAEKK
AKFSGTP-ALSKELNWVEEVT-AEYVSRMARIARKLFDAMDL-GLAEHDGTFRAYRYPAC
-P-IGMEAHTDSSVLSILNQQDESSLQVTWRSVQPEGT--LVVNIGDMMQAITGDAYRSV
EHRV-VP-TDRMSLCYFAFPQDDAVISGCGYRPFSYEFREQ
>HORVU3Hr1G117870.1
LRAVDLEAILTEELAAACRDPGVFRLVN--HGVPSN--LTARLLALARGLL-ELDAATKS
R-FCGTP-ALAKEPNWLEEAVSGEYVAHMARIARKVFDTLASGELTERGCIFRAYRYPAA
-A-VGMEAHTDSSVLSILN-QDVGGLQVCWRAVRPEGA--LVVNVGDMLQAMSGDAYRSP
EHRV-VA-ADRMSLCYFAFPEEEAVIVGGRYQGFSYEFREQ
>LOC_Os04g33360.1
LPTLDLEREDRAALVAACRDLGAFRVVN--HGVPGE--LRRRLLELGKQLLGRDTFELKK
A-FWGTAALKSKEVNWLEALM-AEYGDHMARIARKLFDALAA-ELAERQGFLRLYRYPPC
-P-LGMEPHTDSSVLSIILGQDVGGLQVAWRDVAPPGE--LLVNLGDMMTAISGGSYQSV
RHRV-LASTERVSCCYFAFPQEDAVVEAPSYRPFSYEFREQ
>KQL09066
LPTVDLEAPA---LDAACRGLGIFRLAN--HGVPAD--LTARLFALARDLLGRTPFPEKK
A-FWGTPEEPSRDVNWIEDLV-AEYADHMARVARRLFDAIAE-ALNEHGGYLRVHRYPRC
-P-HGIQAHTDSTTLSIIN-QDSGGLQVAWRDLAADGA--LLVNLGDMAKAISADAYRSV
PHRV-VADDERLSLCYFAFPRDDAVVS---YRPFTVEFRAQ
>KQL09067
VPTVDLQA-----LAAACRRLGVFRLAN--HGVPGD--LSARLFALARDLLGRAPFLDKQ
A-FWGTP-ALSRDVNWVEDLA-REYGAHMARVARALFDALAA-ALAERDGFLRVYRYPPC
-P-LGMEAHTDSSVLSVIN-QDVGGLQVAWRDVAPAGT--LLVNLGDMARAISGDAWRSV
RHRV-AA-AERLSLCYFAFPRDDAVITC--YRPFTYEFREQ
>Zm00001d035994_P001
LPTVDLEA-----LAAACRELGVFRLAN--HGVPAA--LSARLFALARAALAGAPFRDKR
A-FWGTP-ALSRDVNWVEGLARAEYGAHMTRVARTLFDALAP-ALAERDGFLRVYRYPRC
-P-LGMEAHTDSSVLSVIN-QEAGGLQVAWRDVAPAGASTLLVNLGDMARAISGDAYRSV
RHRV-AA-AERLSLCYFAFPRDDAVIT---YRPFTYEFREQ
>OQU85872
LPTVDLDA-----LAAACRGLGVFRLAN--HGVPAS--LSARLFATARAALAGAPFHDKR
A-FWGTP-ALSRDVNWVEDLA-AEYGAHMARVARALFDALAA-PLAERDGFLRVYRYPRC
-P-LGMEAHTDSSVLSVIN-QDVGGLQVAWRDVAPTGT--LLVNLGDMARAISGDAYCSV
RHRV-AA-AERLSLCYFAFPQDDAVIS---YRPFTYEFREQ
>KQK21235
LPVIDLAPACRAAMVRAASEWGFFQVTN--HGVPQP--LLDELHGQQLRAF-RRPFHRKL
APRWGNP-TATHQLSWSERVI-HEVSAAMSKLARRILSVLAA-EHETTTCFLRLNRYPQA
-P-LGLCGHTDSDFLTILR-QDVGGLQLRWRTVRPPGA--LTVNVGDLLQAWTNDVYASV
EHRV-VA-RERFSVAFFLCPSYDTLIRPPLYRNFTFEYRSQ
>LOC_Os07g01340.1
LPVIDLAGVCRADMVRAASEWGFFQVTN--HGVPQA--LLRELHDAQVAVF-RRPFQEKV
TERWGTP-TAKEQLSWSEAVI-EEVSRAMYELAQKLAEILMR-GLREETCFLRLNRYPPC
-A-FGLCPHTDSDLLTIVHQQQVGGLQLRWVAVKPPST--LIVNVGDLLQAWSNDVYKSV

EHRV-MA-LERFSMAFFLCPSYHTLIIPSSYRSFTFEYRKQ
>KQK85900
LPVIDLGSQCRAAIVRAASEWGFFQVTN--HGVPQP--LLDELHDAQVAVF-RRPFERKL
RERWGTP-TATEQLSWSE-VI-EEVSTAMSKLALQLAGILVA-DLTRNTCFLRLNRYPAC
-G-FGLCPHTDSDFLTILH-QDVGGLQLRWVAVKPPGA--LIVNVGDLLQAWSNDRYRSV
EHRV-MA-SERFSVAFFLCPAYDTLIRPRSYRSFTFEYRNQ
>Zm00001d018617_P001
LPVIDLSSQCRASIVRAASEWGFFQVTN--HGVPQV--LLDELHQAQAGVF-RRPFQLKA
HQRWGTP-TATEQLSWSELVV-EEVSTAMSKLAQRLAGILVA-ELTRSTCFLRLNRYPPC
-AVYGLCPHSDSDFLTILH-QDVGGLQLQWVAVKPPGA--LIVNVGDLLQAWSNDRYRSV
EHRV-MA-RERFSAAFFLCPSYDTLIRPRCYESFTFEYRNQ
>EER95722
LPVIDLSSCRAS-IVRAASEWGFFQVTN--HGVPQA--LLDELHQAQVAVF-RRPFHLKA
SQRWGTP-TATDQLSWSELVV-EEVSTAMSKLAQRLAGILVA-DLTRSTCFLRLNRYPPC
-P-YGLCPHTDSDFLTILH-QDVGGLQLRWVAVKPPGA--LIVNVGDLLQAWSNDRYRSV
EHRV-MAARERFSVAFFLCPSYDTLVRPRCYESFTFEYRNQ
>HORVU2Hr1G090030.9
XXXXXXXXXXXXXXXXXXXXXXXXXXXX--XXXXXX--XXXXXXXXQARLF-RLPFDTKE
KARWGNP-TATRHLSWSEYVLTSXXXXXXXXXXXXXXXXXXX-XXXXXXXXLRLNRYPAC
-P-FGLVPHTDSDFLTILC-QDVGGLQLRWVAVKPPDA--LIVNVGDLFQAWSNNRYKSV
EHKV-VA-AERFSVAYFLCPSYDSPVGTCSYRPFTFEYRRK
>LOC_Os02g41954.1
LPMIDVGCCAAA-IAAAAAEWGFFQVVN--HGVAQE--LLEAMRREQARLF-RLPFEAKS
SARWGTP-TATRQLSWSEDVT-REVADAMSRLARALARVLAE-SLDDATCFLRLNRYPPC
-P-FGLVPHTDSDFLTVLC-QDVGGLQLRWVAVKPPGA--LIVNIGDLFQAWSNNRYKSV
EHRV-MT-TERYSVAYFLCPSYDSPIGTCRYKAFTFEYRRR
>KQK00433
LPMIDVGCCAAA-IAGAAEDWGFFQVVN--HGVKQE--LLEAMRREQTRLF-RLPFEAKA
TARWGTP-TATAQLSWSEDVT-QEVATAMSKLANTLARVLAE-RLDERTCFLRLNRYPPC
-P-FGLVPHTDSDFLTVLC-QDVGGLQLRWLAVKPPNA--LIVNIGDLFQAWSNNRYRSV
EHKV-VT-TERYSVAYFLCPSYDSPIGACEYRTFTFEYRRR
>KQL30590
LPMIDVGCCAAA-IARAAEEWGFFQVRN--HGVAPG--LLDAMRREQARLF-RLPFEAKS
TARWGTP-TATRQLSWSEDVT-REVAGAMSKLAGTLARVLAE-ALDETTCFLRLNRYPPC
-P-FGLVPHTDSDFLTVLC-QDVGGLQLRWVAVKPPGA--LIVNIGDLFQAWSNNRYKSV
EHKV-VT-TERYSVAYFLCPSYDSPIGTCEYRTFTFEYRSK
>Zm00001d017294_P001
LPMIDVGCACAAAIARAAEEWGFFQVRN--HGVPQE--LLEEMRREQARLF-RLPFETKA
TARWGTP-TATRQLSWSEDVT-REVAGAMSKLAGTLARVLAE-ALDETTCFLRLNRYPAC
-P-LGLVPHTDSDFLTVLC-QDVGGLQLSWVAVKPPGT--LVVNIGDLFQAWSNNRYKSV
EHKV-MT-TERYSVAYFLCPSYDSPIGT--YRTFTFEYRRK
>KXG30679
LPMIDVGCACTAAIARAAEEWGFFQVRN--HGVSQE--LLDEMRREQARLF-RLPFEAKA
TARWGTP-TATQQLSWSEDVT-REVAGAMSKLAGTLARVLAE-EADETTCFLRLNRYPPC

-P-FGLVPHTDSDFLTVLC-QDVGGLQLRWVAVKPPGA--LIVNIGDLFQAWSNNRYKSV
EHKV-MT-TERYSVAYFLCPSYDSPIGT--YRTFTFEYRRK
>HORVU4Hr1G072540.2
--------ACADAMARAASEWGFFQVVN--HGVGLE--LLEEMRREQAKLF-RLPFGTKD
KARWGNP-TATGQLSWSEGVM-QEVADAMSRVADTVAGTLAE-NLDGTTCFLRLNRYPAC
-P-LGMVPHTDSDFLTILF-QDVGGLQLRWVAVKPAEA--LIVNVGDLFQAWSNNKYKSV
EHKV-VA-AERFSVAYFLCPSWDSPVGT--YKPFTFEYRRS
>KQK98136
LPLIDLGCACAYAMARAASEWGFFQVTG--HGVGRA--LLERLRAEQARLF-RLPFETKA
RARWGAP-TATRHLSWSEGVM-QEVADAMSRVAKTVAVALAG-SLDETTCFLRLNRYPAC
-P-FGLVPHTDSDFLTVLC-QDVGGLQLRWVAVKPPDA--LIVNIGDLFQAWSNNRYKSV
EHKV-VA-AERFSAAYFLCPSYDSPVGT--YRSFTFEYRRM
>Zm00001d002999_P001
LPLIDLTCCADA-MARAASEWGFFQVTG--HGVSRA--LLERLRAEQARLF-RLPFETKA
KARWGAP-TATRHLSWSEDVV-QEVADAMSRVAKTVAVALAGSLLGETTCYLRLNRYPAC
-P-FGLVPHTDSDFLTVLS-QDVGGLQLGWVAVKPPDA--LIVNIGDLFQAWSNNLYKSV
EHKV-VA-AERFSAAYFLCPSYDSLVGT--YRDFTFEYRRK
>KXG26736
LPLIDLSCCADA-MARAASEWGFFQVTG--HGVSRA--LLERLRAEQARLF-RLPFETKA
KARWGAP-TAARHLSWSEGVM-QEVADAMSRVAKTVAVALAG-SLDETTCYLRLNRYPAC
-P-FGLVPHTDSDFLTVLC-QDVGGLQLRWVAVKPPDA--LIVNIGDLFQAWSNNRYKSV
EHKV-VA-AERFSAAYFLCPSYDSPVGT--YRDFTFEYRRK
>LOC_Os04g44150.1
LPMVDLERACAGAMARAASEWGFFQLTN--HGVGRE--LMEEMRREQARLF-RLPFETKE
KARWGNP-TATRHLSWSEGVM-QEVAEAMSRVANTVAAALAE-ELDETTCFLRLNRYPAC
-P-FGLVPHTDSDFLTVLC-QDVGGLHLRWVAVRPPDA--LVVNIGDLFQAWSNNRYKSV
EHKV-VA-TDRLSVAYFLCPSYDSLVGT--YRAFTFEYRKK
>KQJ83649
LPMIDIECCADA-MARAASEWGFFQVTN--HGVGKE--LLEEMRKEQARLF-RLPFETKR
KARWGNP-TATRQLSWSEGVM-QEVADAMSRVADAVAGALAE-KLDGTTCFLRLNRYPAC
-P-FGLVPHTDSDFLTILC-QDVGGLQLRWVAVKPPDA--LIVNIGDLFQAWSNNRYKSV
EHKV-VA-AERFSVAYFLCPSYDAPVGT--YRSFTFEYRRK
>HORVU2Hr1G118350.1
VPVVDMRDAAEA-VARASEQWGAFLLEG--HGVPTE--LLARVEAGIAGMF-ALPTPEKM
RAGYGPP-LIASKNMWSEGVM-EEFLKEMRSLANRLMELFLV-ALESMTETMHLNWYPKC
-P-LGLKGHTDSGFFTFVM-QSVPGLHLRWVEVPAPGA--LIVNIGDLFQILTNGRFRSV
YHRV-VV-RERISVAYFLIPPADVKVAPLKYRALTWEAFAN
>HORVU3Hr1G022840.8
VPVVDMRDAAEA-VALAAQDWGAFLLQG--HGVPLE--LLARVEAAIAGMF-ALPASEKM
RAGYGSP-PISSKCMWSEAVM-EEFHREMRVLADKLLELFLV-ALETMTATMHLNWYPKC
-P-LGLIAHTDSGFFTFVL-QSVPGLQLRWVTVPAPGA--MVVNVGDLFHILTNGRFHSV
YHRA-VV-SDRISLGYFLGPPAHVKVAPLRYRAVTWGVRKK
>LOC_Os01g08220.1
VPVVDVGAAAAR-VARAAEQWGAFLLVG--HGVPAA--LLSRVEERVARVF-SLPASEKM

RAGYGSP-PISSKLMWSEDVM-EEFHKEMRRLADELLRLFLR-ALERMTATVHLNWYPRC
-P-LGLIAHTDSGFFTFVL-QSVPGLQLRWVAVPAAGA--FVVNVGDLFHILTNGRFHSV
YHRA-VV-RDRVSLGYFLGPPPDAEVAPLPYRAVTWAVRKK
>KQL04660
VPVVDLREAAVARVARAAEQWGAFLLTG--HGVPAE--LLARVEDRVACMF-ALPAADKM
RAGYGSP-PISSKCMWSEDVM-EEFHKEMRALADRLLELFLR-ALETMTATMHLNWYPRC
-P-LGLIAHTDSGFFTFVL-QSVPGLQLRWVAVPAPGA--FVVNVGDLFHILTNGRFHSV
YHRA-VV-LDRISLGYFLGPPPHAKVAPLRYRAVTWGVRKK
>Zm00001d039634_P001
VPVVDLGAAPAAAVARAAEQWGAFLLTG--HGVPAD--LLARVEDRIATMF-ALPADDKM
RAGYGSP-PISSKCMWSEDVM-EEFHKHMRALADKLLELFLM-ALETMTATMHLNWYPRC
-P-LGLIAHTDSGFFTFVM-QSVPGLQLRWVAVPAPGA--FVVNVGDLFHILTNGRFHSV
YHRA-VV-LDRISLGYFLGPPPHAKVAPLRYRAVTWGVRKK
>EES00202
VPVVDLAGAVVAQVARAAEQWGAFLLTG--HGVPAE--LLARVEDRIATMF-ALPADDKM
RAGYGSP-PISSKCMWSEDVM-EEFHKHMRALADKLLELFLM-ALETMTATMHLNWYPRC
-P-LGLIAHTDSGFFTFVL-QSVPGLQLRWVAVPAPGA--FVVNVGDLFHILTNGRFHSV
YHRA-VV-LDRISLGYFLGPPPHAKVAPLRYRAVTWGVRKK
>KQJ89115
VPVVDLRDVAAA-MARAAEQWGAFLLLG--HGVPAD--LVARVEERIEAMF-ALPASEKT
RAGYGSP-PISDKSMWSEEVM-EEFHKGMFALAHKLLGLFLS-ALDTMAASMHLNWYPKC
-P-VGLIAHTDSGYFTFVL-QSVPGLQLRWVAVPAPGA--FVINVGDLFNIVTNGRFHNV
FHRA-VV-SHRVSLGYFLGPPAQAVVAPLDYRPVVWGLREK
>KQK02988
VPVVDMRAAAAAAVARAAETWGAFLLEG--HGVPES--LLARVEERVAGMF-ALPAPEKM
RAGYGSP-PISAKSMWSEEVM-EEFHREMRLLADKLLGLFLR-ALETMTATTHLNWYPRC
-P-LGLIPHTDSGYFTFVL-QSVPGLQLRWVAVPAPGA--FVVNVGDLFSILTNGRFHSV
FHRA-VV-SHRVSLGYFLGPPAQTRVGPLEYRPVTWTVRKK
>LOC_Os05g08540.1
VPVVDMSGAVAA-VARAAEEWGGFLLVG--HGVTAE--ALARVEAQAARLF-ALPADDKA
RGGYGVPPYLL-KQMWAESAM-EEYDSSMRALGERLLAMFFK-ALETLTSTIHLNMFPRC
-P-VGLAAHTDSGFFTFIL-QSVPGLQLRWVTVPGPGA--LIVVVGDLFHVLTNGRFHSV
FHRA-VV-RDRISMPYFLGPPADMKVTPLVYQAVTWAVRDK
>KQL13797
VPVVDLGGVARAAAGRAAEEWGAFLLVG--HGVAAG--VAARVEEQVARLF-ALPAAEKA
RAGYGRAPRLNSNHMWSEDVM-EEHHAEMRALGVRLLDMLFG-ALETLTATTHLNMYPRC
-P-IGMAAHTDSGFITIIL-QSVPGLQLRWVTVPAPGA--LVVVLGDLFQVLTNGRFRSA
LHRA-VV-RDRISVPYFLGPPADMKVAPLAFRGVTWEIREK
>Zm00001d037627_P001
VPVVDMGVATRA-LARAADEWGVFLLVG--HGVPRE--VAARAEEQVARLF-VLPAPDKA
RAGYGRP-PLASKLMWSEDVM-EEYDREMRALGGRLLDLFFM-ALETWTATMHPILYPRC
-P-IGLTAHTDSGFITLIM-QSVPGLQLRWVTVPAPGA--LIVMLGDLFQVLTNGRFRSP
IHRA-VV-RERISVPYFLCPPEDMTVAPLAFRAVTWEVKHK
>KXG21453

VPVVDMGMAARA-VARAAEEWGAFLLVG--HGVPRG--VAARAEAQVARLF-ALPAPDKA
RAGYGMP-PLASKLMWSEDVM-EEYDREMRALGGRLLDLFFM-ALDNLTAMMHPILYPKC
-P-MGLAPHTDSGFITLIT-QSVPGLQLRWVTVPAPGA--FVVVLGDLFQVLTNGRYRSA
LHRA-VV-RDRISVPYFLGPPDGMKVAPLAFRAVTWELKHK
>KQK10911
MPVVDVGVAVAQ-VAAAGATHGFFQVSG--HGVDAA--LARAALDGASDFF-RMPLADKQ
RAGYTSA-HADTKLPWKERVY-QEYCEKMKELSLTIMELLEL-SLADSSSIMRCNYYPPC
-P-LGTGPHCDPTALTILL-QDVGGLEVDWRPVRPPGA--MVINIGDTFMALSNGRYKSC
LHRA-VV-QERRSLAFFLCPRQDRVVRPPLYPDFTWRFTQR
>HORVU3Hr1G090980.3
VPVVDVGVAVAQ-VASACATHGFFQVSG--HGVDNA--LARAALDGASGFF-RLPLAEKQ
RAGYTSA-HADSKLPWKERVY-QEYCGKMKELSLRIMELLEL-SLADSSSIMRCNYYPPC
-P-LGTGPHCDPTALTILL-QDVGGLEVDWRPVRPPGA--MVINIGDTFMALSNGRYKSC
LHRA-VV-QERRSLAFFLCPREDRVVRPPPYPDFTWRFTQR
>LOC_Os01g66100.1
MPVVDVGVAAAQ-VAAACATHGFFQVSE--HGVDAA--LARAALDGASDFF-RLPLAEKR
RAGYTSA-HADSKLPWKERVY-QKYCEEMKELSLTIMELLEL-SLADSSSIMRCNYYPPC
-P-LGTGPHCDPTALTILL-QDVGGLEVEWRPVSPPGA--MVINIGDTFMALSNGRYKSC
LHRA-VV-RERRSLAFFLCPREDRVVRPPPYPDFTWRFTQR
>KQL08016
VPLVDVGVAAAQ-VAAACATHGFFQVCG--HGVGAD--LARAALDGASDFF-RLPLAEKQ
RAGYTSA-HADSKLPWKERVY-QRYCEEMKALSLTIMELLEL-SLEDSRSIMRCNYYPPC
-P-LGTGPHCDPTALTILL-QDVGGLEVDWRPVRPPGA--MVINIGDTFMALSNGRYKSC
LHRA-VV-QERRSLAFFLCPREDRVVRPPAYPDFTWRFTQR
>Zm00001d012212_P001
VPMVDVGVAAAQ-VAAACATHGFFQVCG--HGVDAA--LGRAALDGASDFF-RLPLAEKQ
RAGYTSA-HADAKLPWKEWVY-QRYCEEMKELSLTIMELLEL-SLEDSRSIMRCNYYPPC
-P-LGTGPHCDPTALTILL-QDVGGLEVEWRPVRPPGA--MVINIGDTFMALSNGRYKSC
LHRA-VV-RARRSLAFFLCPREDRVVRPPAYPDFTWRFTQR
>Zm00001d042611_P001
VPVVDVGVAAAQ-VAAACATHGFFQVCG--HGVDAA--LGRAALDGASDFF-RLPLAEKQ
RAGYTSA-HADSKLPWKERVY-QRYCEEMKELSLTIMELLEL-SLEDSRSIMRCNYYPPC
-P-LGTGPHCDPTALTILL-QDVGGLEVEWRPVRPPGA--MVINIGDTFMALSNGRYKSC
LHRA-VV-QERQSLAFFLCPREDRVVRPPAYPDFTWRFTQR
>OQU87995
VPVVDVGVAAAQ-VASACATHGFFQVCG--HGVDAA--LGRAALDGASDFF-RLPLADKQ
RAGYTSA-HADSKLPWKERVY-QRYCEKMKELSLTIMELLEL-SLEDSRSIMRCNYYPPC
-P-LGTGPHCDPTALTILL-QDVGGLEVEWRPVRPPGA--MVINIGDTFMALSNGRYKSC
LHRA-VV-QERRSLAFFLCPREDRVVRPPAYPDFTWRFTQR
>LOC_Os05g34854.1
VPVVDLAAAVAQ-VAAACASHGFFQVSG--HGVPPS--LARAALDGAAGFF-RLPPAAKQ
RAGYTAA-HADDNLPWKEEVY-QEYCEAMEEVTKAIMAVLGE-SLEDSSSIMRCNYYPPC
-P-LGTGPHCDPSALTVLL-QDVDGLQVAWRPVRPPGA--FVVNIGDTFMALTNGRYKSC
LHRA-VV-QERRSLAFFLCPREDRVVRPPAYPDFTWRFTQR

>EES19547
APVVDVGAAAEL-VSGACSSHGLFQVTG--HGLDPA--LARAALDGAAAFF-RLPLATKQ
RAGYTAA-HADANLPWKEGVVYQNYCNAMKEVSLAIMEVIGV-SLADGSSIMRCNYYPPC
-P-LGTGPHCDPSALTVLL-QDVDGLQVEWRTVRPPGA--LVVSIGDTFMALSNGRYRSC
LHRA-VV-RERRSLVFFLCPREDRVVRPPPYPDFTWRFTQR
>KQK06181
VPVVDLAAAAAQ-AAAACASHGFFLVTG--HGVDPA--LARAALDGAAGFF-RLPLATKQ
RAGYAAA-HADANLPWKEEVY-QEYCGAMEAASLAIMEVLGV-SLADGSSVMRCNYYPPC
-P-LGTGPHCDPSALTLLM-QDVDGLQVGWRPVRPPDE--LVVNIGDTFMALSNGRYKSC
LHRA-VV-RERRSLAYFLCPRKDRVVRPPPYPDFTWRFTQR
>HORVU1Hr1G063780.2
VPVVDIGAAAAQ-VAEACASHGFFQVTG--HGVDPA--LAQAALDGAADFF-RLPLATKQ
RAGYASA-HADAKLPWKEEVY-QEYCEAMEDASLAIMEVLGV-SLADGSSIMRCNYYPRC
-P-LGTGPHCDPSALTILL-QDVDGLQVAWRSVRPPGE--LVVNIGDTFMALSNGRYKSC
LHRA-VV-KERRSLAYFLAPQEDRVVRPPPYPDFTWRFTQR
>HORVU3Hr1G089980.1
VPVVDLAGAGLNELVAACERHGFFQVVN--HGVDPA--LLAKAYRCCDAFY-ALPLAEKQ
RAGYAGS-FVGSKLPWKEDVW-QEYCNEMTRLALDVTEVLAA-CLAGDDSLMRLNHYPPC
-K-LGTGPHHDPTALTLLH-QDVGGLEVAWRAVRPSDA--FVVNIGDTFSALTNGRHVSC
LHRA-VV-LARRSLTFFLNPQLDRPVTPPAYPDFTWEFTQK
>KQL22579
IPVVDLAELPVG-MAEACERHGFFQVVG--HGVGAE--LIAEAYRCCDAFY-AHPLAEKQ
RAGYASS-FTGSRLPWKEEVY-QEYCDAMTRLALDVTEVLAA-ALAGGDSVMRLNHYPPC
-R-LGTGPHRDPTSLTLLH-QDVGGLQVEWLAVRPADA--FVVNIGDTFAALTDGRHASC
LHRA-VV-AARRSLAFFLNPPLDRVVCPPEYPDFKWEFTQK
>Zm00001d007894_P001
IPVVDLGELPRG-VAEACERHGVFQVVN--HGVGAA--LLAEAYRCCDAFY-ALPLADKQ
RAGYASS-FTGCCLPWKEEVY-QEYCDAMTRLALDVTEVLAA-ALEGGDSVMRLNHYPAC
-R-LGTGPHRDPTSLTLLH-QDVGGLQVPWRAVRPADA--FVVNIGDTFAALTDGRHTSC
LHRA-VV-GSRRSLAFFLNPPLDRVVRPPGFPDFTWEFTQK
>EER95912
IPVVDLGELPRG-VAEACERHGFFQVVN--HGVPAA--LLADAYRCLDAFY-ARPLADKQ
RAGYASS-FTGSCLPWKEEVY-QEYSDEMARLAMDVTEVLAA-ALDGGDSIMRLNHYPAC
-R-LGTGPHRDPTSLTLLH-QDVGGLQVEWRAVRPADA--FVVNIGDTFAALTDGRHASC
LHRA-VV-RARRSLAFFFNPPLDRVVRPPDFPDFTWEFTQK
>LOC_Os07g07420.1
IPVVDLAAIGRD-VAEACERHGFFQVVN--HGVDPA--LLAEAYRCCDAFY-ARPLAEKQ
RAGYASS-FTGCKLPWKEEVY-QEYCDVMTRLALDVTEVLAV-ALADGDPVMRLNHYPPC
-R-LGTGPHRDPTSLTLLH-QDVGGLQVGWRAVRPADA--FVVNIGDTFAALTNGRHASC
LHRA-VV-VARRSLTFFLNPRLDRVVSPPPFPDFTWEFTQR
>KQK20711
IPVIDLAAGEDH-FAAACERHGFFQIVN--HGVDPS--LLAEAYRCMDAFY-ALPLAEKQ
RAGYAGS-FTGSRLPWKEEVW-QGYCDVMTRLAQDVTDLLAV-GLAGGDSVMRLNNYPPC
-R-LGTGPHRDPTSLTLLH-QDVGGLQVEWRAVRPEDA--FVVNIGDTFAALVDGRHASC

LHRA-VV-AARRSLTFFLNPPLDRVVSPPPYPDFTWEFTQK
>KQK20710
IPVIDLAAGADH-FAAACERHGFFQIVN--HGVDPA--LLAEAYRSMDAFY-ALPLAEKQ
RAGYAGS-FTGSRLPWKEEVW-QEYCDVMTRLALDVTDLLAV-GLAGGDSVMRLNNYPPC
-R-LGTGPHRDPTSLTLLH-QDVGGLQVEWRAVRPEDA--FVVNIGDTFAALVDGRHASC
LHRA-VV-AARRSLTFFLNPPLDRVVSPPQYPGFTWEFTQK
>LOC_Os03g63970.1
VALIDVGAVVRQ-VGEACERHGFFLVVN--HGIEAA--LLEEAHRCMDAFF-TLPLGEKQ
RAGYASS-FTGSKLPWKEEVY-SRYCHEMSRLSLELMEVLGE-SLQRNDSIMRLNYYPAC
-Q-LGTGPHCDPTSLTILH-QDVGGLEVRWRAIRPPGA--LVVNVGDTFMALSNARYRSC
LHRA-VV-APRRSLAFFLCPEMDTVVRPPEYPDFTWDFTQR
>KQK86097
VPLIDLSGVVRQ-VGDACGLHGFFQVVN--HGIDPA--LLAEAHRCMDTFF-TLPLADKQ
RAGYASS-FTGSKLPWKEEVY-GRYCSEMSRLSLEIMEVLGE-SLEGNDSIMRLNYYPPC
-Q-LGTGPHCDPTSLTILH-QDVGGLQVRWRSIRPAGA--FVVNIGDTFMALSNGRYRSC
LHRA-VV-VPRRSLAFFLCPEMDKVVRPPGYPDFTWDFTMR
>Zm00001d034898_P001
VPLIDLSGVVRQ-VRRACDLHGFFQVVG--HGIDAA--LTAEAHRCMDAFF-TLPLPDKQ
RAGYASS-FTGSKLPWKEEVY-GRYCSEMSRLSLELMEVLGE-SLQGNDSIMRLNYYPPC
-Q-LGTGPHCDPTSLTILH-QDVGGLQVAWRSIRPPGA--FVVNIGDTFMALSNGRYRSC
LHRA-VV-VARRSLAFFLCPEMDKVVRPPKYPDFTWDFTMR
>EER90481
VPLIDLSGVVRQ-VRRACDLHGFFQVVN--HGIDDA--LLQEAHRCMDAFF-TLPMSDKQ
RAGYASS-FTGSKLPWKEEVY-GRYCSEMSRLSLELMEVLGE-SLQGNDSIMRLNYYPPC
-Q-LGTGPHCDPTSLTILH-QDVGGLQVRWRSIRPPGA--FVVNIGDTFMALSNGRYRSC
LHRA-VV-VPRRSLAFFLCPEMDKVVRPPAYPDFTWDFTMR
>KQK12006
VPLIDIGGSVARLVGDACSRHGFFQVVN--HGIDAA--LLADAHRCVDAFF-KLPLAEKQ
RAGYASS-FVGSKLPWKEEVY-ARYCEEMSRVSLEIMEVLGE-SLEGNDSIMRLNYYPPC
-Q-LGTGPHCDPTSLTILH-QDVGGLQVRWRAIAPQDA--FVVNIGDTFMALSNGRYKSC
LHRA-VV-VPRKSLAFFLCPEMDKTVAPPGYPDFTWDFTQK
>HORVU5Hr1G124120.2
VPLIDIGGVTRL-VGEACERHGFFQVVN--HGIDAQ--LLADAHRCVDAFF-TMPLPEKQ
RAGYASS-FTGSKLPWKEEVY-ARYCSEMSRLSLEIMEVLGE-SLEGNESIMRLNYYPPC
-Q-LGTGPHCDPTSLTILH-QDVGGLQVRWRSIRPADA--FVVNIGDTFMALSNGRYKSC
LHRA-VV-VPRKSLAFFLCPEMDKVVAPPGYPDFTWDFTQK
>LOC_Os01g22910.1
SGDGDSGRQRRL-IRRWGGLWRWIRRRG---GAAAA--CEEEVEGRRRIRRWGLRWLARR
SSGAAADPAMGTAFPLALQVV-NDYVEAVRQLACHVLDLLGE-GLTDNDSLIRINHYPPS
CA-IGFGEHTDPQILSVLR-ANADGLQLVWVPVPPPSA--FFVNVGDLLQALTNGRLVSI
RHRV-VV-KPRLSTIYFAAPPLHARISALPYRAFTWEYKRT
>LOC_Os05g06670.1
IATVDMSAVARQ-VATACAAHGFFRCVG--HGVPAAAPVAARLDAATAAFF-AMAPAEKQ
RAGYGCR-SIGGDVGELESAIVNEYIEAMKKLACEILDLLGE-GLADSDCLLRINHYPPS

-C-IGFGEHSDPQILSLLR-ANVEGLQVMWVQVPSPSA--IFVNVGDVLQALTNGRLISI
RHRV-IA-RPRLSTIYFASPPLHARISALPYRSFTWEYKTT
>KQK99291

------------------TVV-NEYVGAVRQLACEILDLLGE-GLTDSDSLLRINHYPPA
-C-IGFGEHSDPQIISLLR-ANVNGLQVVWIKVPAPSA--FFVNVGDLLQALTNGKLVSV
RHRV-IA-RPRLSTIYFAAPPLHAQISALPYRSFTWEYKKT
>Zm00001d024175_P001
IPTVDLSALSRQ-VVRACAEHGFFRAVN--HGVPPG--PAARLDAAARTFF-ALAPRDKQ
RAGYGCR-SIGGDAGELENVV-NEYVGAMRQLACEILDLLGE-GLTDSDSLLRINHYPTA
-C-VGFGEHSDPQILSLLR-ANVDGLQVVWVQVPAQSA--FFVNVGDLLQALTNGKLVSV
RHRV-IA-RARLSTIYFAAPPLHARILALAYRPFTWEYKKT
>Zm00001d037565_P002
IPTVDLSALSRQ-VARACAQHGFFRAVN--HGVAPG--PAARLDAAARTFF-ALAPHNKQ
RAGYGCR-SIGGDAGELENVV-NEYVGAMRQLACEILDLLGE-GLTDSDSLLRINHYPPP
-C-IGFGEHSDPQILSLLR-SNVDGLQVVWVQVPAPSA--FFVNVGDLLQAFTNGKVISV
RHRV-IA-RARLSTIYFAAPPLHARILALPYRPFTWEYKKT
>OQU77483
IPTVDMSALSRQ-VARACAEHGFFRAVN--HGVAAG--PAARLDAAARTFF-ALPPHDKQ
RAGYGCR-TIGGDAGELENVV-NEYVGAMRQLACEILDLLGE-GLTDSDSLLRINHYPPA
-C-IGFGEHSDPQILSLLR-SNVDGLQVVWVQVPSPSA--FFVNVGDLLQALTNGKVISV
RHRV-IA-RARLSTIYFAAPPLHARILALPYRPFTWEYKKT
>KQK07366
IPTVDMSALSRQ-VARACAEQGFFRAVN--HGVPAP--AARQLDAATAAFF-ALPAHEKQ
RAGYGCR-TIGGDVGELESCVVNEYVEAVKQLACDILDLLGE-GLPDSDSLLRINHYPPS
-C-IGFGEHSDPQILSLLR-ANVDGLQVVWIQVPASSA--FFVNVGDLLQALTNGKLLSV
RHRV-IA-RPRLSTIYFAAPPLHARISALPYRSFTWEYKTT
>HORVU1Hr1G023460.3
IPTVDMSALSRQ-VARACAEQGFFRAVN--HGVPAG--PPARLDAATSAFF-ALAAHDKQ
RAGYGCR-SIGGDVGELESVV-NEYVEAVKQLACDILDLLGE-GLADSDSLLRINHYPTK
AG-IGFGEHSDPQLLSLLR-ANVDGLQVAWIQVPASSA--YFVNVGDLLQALTNGRLVSI
RHRV-IA-RPRLSTIYFAAPPLHARISALPYRSFTWEYKTA
>KQL05037
IPTVDMSALARR-LVRACAERGFFKAVN--HGVPAR--AAARLDAAASAFF-ARPARAKQ
AAGYGSR-NIGGDVGELESVAVNEYVDAVRRLACRVLDLLGE-GLVDSDSLLRINHYATS
AA-IGFGEHTDPQILSVLR-ANVDGLQVEWVPVPAPAA--FFINVGDLLQALTNGRLVSI
RHRV-MA-RPRLSTIYFAAPPLRALIAALPYRPFTWEYKKA
>Zm00001d040737_P001
IPTVDMSALARR-LVRACAERGFFKAVN--HGVPPR--VSARLDAATSAFF-ARPAPVKQ
AAGYGSR-NIGGDVGELESVAVNEYVDAVRRLACRVLDLLGE-GLVDSDSVLRINHYPTS
-S-IGFGEHTDPQILSLLR-ANVDGLQVQWVQVPAPSA--FFINVGDALQALTNGRLVSI
RHRV-MA-RPRLSTIYFAAPPLDARIAALPYRTFTWEYKKA
>EES02896
IPTVDMSALARR-LVRACAERGFFKAVN--HGVPPR--VSARMDAAASAFF-ARPGQAKQ

AAGYGSR-NIGGDVGELESVAVNEYVGAVRHLACRVLDLLGE-GLVDSDSLLRINHYPTS
RS-IGFGEHTDPQILSLLR-ANVDGLQVQWVQVPAPSA--FFINVGDLLQALTNGRLVSI
RHRV-MA-RPRLSTIYFAAPPLDARVAALPYRTFTWEYKKA
>KQK04223
IPTVDMSALSRQ-MVEAFAERGFFKAVN--HGVPPR--APATLDAATAAFF-ERPAPEKQ
AAGYGSR-SIGGDVGELEEAV-NEYVGAVRRLACQILDLLGE-GLSDSDSIIRINHYPPS
SA-VGFGEHSDPQILSVLR-ANVDGLQVTWVQVPAPAA--FFINVGDLLQALTNGRLVSI
RHRV-MA-KPRLSTIYFAAPALHALISALPYRPFTWEYKKN
>HORVU7Hr1G101720.1
IPTIDMSAMSRQ-MVEAFAERGFFKAVN--HGVPPR--ASARLDAASAAFF-ARPAEEKQ
EAGYGSR-SIGGDVGELEEAV-NEYVQAVRHLACRILDMLGE-GLTDGDSLVRINHYPAS
AA-VGFGEHTDPQILSVLR-ANVDGLQVAWVQVPAPAA--FFINVGDLLQALTNGKLVSI
RHRV-MA-KPRLSAIYFAAPALHERISAFPYRPFTWEYKKT
>HORVU3Hr1G029010.7
AGLLQGDRAAGARARRGGGRGGVLRAAAGREGGRRR--PAARVRQQADRRL-RRPRVDR-
--RRAAR-RLLAVRGRRRDLL-EEYAAAVRRMACGVLELMAE-GLGESDNMLRVNHYPPR
-P-TGFGEHTDPQIISVLR-SNTSGLEIEWTSVPPPDA--FFVNVADALQVLTNGRFSSV
KHRG------------------------------WW-----
>LOC_Os05g11810.1
QPEKEAAAAAAA--
--GYGSK-RIGGDLGWVEDIL-NEYVVAVRAMMWEVLKLMAE-GLEESDSVLRVNHYPPH
-P-TGFGEHTDPQIISVLR-SNTSGLEISWASVPPRKS--FFVNVGDVLQ--PGGRIKGF
LHAV-SL-RPAAGLLSLASPTSPPPEQPPAFYPLQHNRRRG
>KQK03260
VQAVDLSSAARA-LVAACEEQGFFKVTG--HGVAPA--LVRALDAAAAAFF-ALPQAEKE
AAGYASK-RIGGDLGWIEGLL-EEYAAALRRMACEVLELMAE-GLGGSDNMLRVNHYPPR
-P-TGFGEHTDPQIISVLR-SNTSGLEIAWASVPPADS--FFINVGDVLQVLTNGRFRSV
KHRV-VV-RPRMSMIFFGGPPPGERLAPLRYREFTWEYKST
>LOC_Os01g11150.1
VRAIDLSAEARA-LVAACEEQGFFRVTG--HGVPPG--LVRAAEAAAARFF-ALPQPDKE
AAGYASK-RIGGDLGWIEELL-REYSAAVRRVACGVLELMAE-GLEDSDSILRVNHYPPR
-P-TGFGEHTDPQIISVLR-SNAPGLEIAWASVPHGDGDSFFVNVGDTLQVLTNGRFRSV
KHRV-VV-KSRVSMVFFGGPPPGERLAPLPYREFTWEYKGS
>KQL04928
VRSVDLSAAARA-LVAACEEHGFFRVTG--HGVPVE--LVRSAEAAAAGFF-ALPQGVKE
E-GYGSK-QIGGDLGWIEDLL-DEYTVAVRRMACAVLELMEE-GLEDSDCVLRVNHYPPR
-P-TGFGEHTDPQIISVLR-SNTSGLEIAWASVPPGDA--FFVNVGDTLQVLTNGRFRSV
RHRV-VV-RSRVSMIFFGGPPPGERLAPLPYLEFTWEFKTS
>Zm00001d008909_P002
VRSVDLSAAARA-LVAACEEHGFFRVTG--HGVPAR--LVRAAEAAAAGFF-ARPQGEKE
EEGYGSK-RIGGDLGWVEDLL-DEYTVAVRRMACAVLELMAE-GLADSDCMLRVNHYPPR
-P-TGFGEHTDPQIISVLR-ANTSGLEIAWASVPPGDA--FFVNVGDTLQVLTNGRFRSV
RHRV-VV-KSRVSMVFFGGPPPGERLGPLPYRDFTWEFKTS
>Zm00001d039394_P001

VRSVDLSAAARA-LVAACEEHGFFRVTG--HGVPAE--LVRAAEAAAAGFF-ARPQGEKD
G-GYGSK-RIGGDLGWVEGLL-DEYTVAVRRMACAVLELMAE-GLADSDCMLRVNHYPPR
-P-TGFGEHTDPQIISVLR-ANTSGLEVAWASVPPGDA--FFVNVGDTLQVLTNGRFRSV
RHRV-VV-KSRVSMIFFGGPPPDERLAPLPYRDFTWEFKTS
>EES00081
VRSVDLSAAARA-LVAACEEHGFFRVTG--HGVPPE--LVRAAEAAAAGFF-AQPQDEKD
EEGYGSK-RIGGDLGWVEDLL-DEYTVAVRRMACAVLELMAE-GLADSDCMLRVNHYPPR
-P-TGFGEHTDPQIISVLR-ANTSGLEIAWASVPPGDG--FFVNVGDTLQVLTNGRFRSV
RHRV-VV-KSRVSMVFFGGPPPGERLAPLP-----------
>Zm00001d037724_P002
VPEVDLAAAARA-VAKACEDHGFFKVTG--HGVPPH--LLARLEAAAAAFF-ALPQREKD
R-GYASK-RIGGDLGWVEAVL-DEYVAAVRRMTCTVLQLMAQ-GLRDSDSMLRVNHYPPA
-A-TGFGEHTDPQIISVLR-SNASGLEITWVSVPSTES--FFVNVGDALQVLTNGRFRSV
RHRV-MV-RPRVSVIFFGGPPPRERLAPLPYREFTWEYKNS
>KXG21531
VPEVDLSAAARA-VAKACEDHGFFKVTG--HGVPAP--LLARLEAAAAAFF-ALPQRDKD
KAGYASK-RIGGDLGWVEDVL-DEYIAAVRRMTCTVLELMAQ-GLRDSDSMLRVNHYPPR
-P-TGFGEHTDPQIISVLR-SNTAGLEISWVSVPSTQS--FFVNVGDALQVLTNGRFRSV
RHRV-MV-RPRVSVIFFGGPPPRERLAPLPYREFTWEYKTS
>HORVU1Hr1G029520.3
LPEVDLSVAARA-VAAACEEHGFFKVTG--HGVPAE--LLARVENAAAAFF-ALSQRDKE
AAGYASK-RIGGDLGWVEDLL-NEYTVAVRRMTCQLLELMAE-GLKESDSMLRVNHYPPR
-P-TGFGEHTDPQIISVLR-SNTSGLEIDWVSVPPQTS--FFVNVGDALQVLTNGRFRSV
RHRV-MV-QPRVSVIFFGGPPPRETLAPLPYKEFTWEYKAS
>KQK07053
VPEIDLAAAAKA-VAAACEEHGFFKVTG--HGVASG--LLARVEAAAADFF-ALPQREKE
AAGYASK-RIGGDLGWVEELL-DEYTVAVRRMTCEVLELMAE-ALEDSDSMLRVNHYPPA
-P-TGFGEHTDPQIISVLR-SNTSGLEITWVSVPPHTS--FFVNVGDALQVLTNGRFRSV
RHRV-MV-RSRVSVIFFGGPAPGKTLAPLPYREFTWEYKAS
>KQL07047
VPVVDLSGAARA-IVDACERFGFFKVVN--HGVAAA--TMDRAETEAIRFF-AQAQADKD
RAGYGSK-RIGGDMGWLEAAL-NEYIAAVRKVAVRVLEAMAE-GLQGSDQVFRVNHYPPC
-P-TGFGEHTDPQLVSVLR-SNTSGLQIQWVSVPSRDA--FFVNVGDSLQVLTNGRFKSV
KHRV-VT-KSRVSFIYFGGPPLAQRIEPLPYKEFTWEYKKA
>Zm00001d012712_P001
VPVVDLSGAARA-IVDACERFGFFKVVN--HGVPAA--TMDVAESEAVGFF-AQAQADKD
RAGYGSK-RIGGDMGWLESAL-NEYVAAVRDVAARVLEAMAE-GLAGSDQVFRVNHYPPC
-P-TGFGEHTDPQIISVLR-SNTSGLQVQWVSVPSRDA--FFVNVGDSLQVLTNGRFESV
KHRV-VT-KSRVSFIYFAGPALEQRIAPLAYREFTWEYKTA
>Zm00001d043411_P001
VPVVDLSSAARA-IVDACERFGFFKVVN--HGVAAA--TMDRAESEAVRFF-AQAQADKD
RAGYGSK-RIGGDMGWLESAL-NEYVAAVRKVAARVLEAMAE-GLAGSDQVFRVNHYPPC
-P-TGFGEHTDPQIISVLR-SNTSGLQIQWVSVPSRDA--FFVNVGDSLQVLTNGRFRSV
KHRV-VT-KSRVSFIYFAGPPLGQRIAPLPYKEFTWEYKKA

>EES01498
VPVVDLSSAARA-IVDACERFGFFKVVN--HGVPAA--TMGRAESEAVRFF-AQAQADKD
RAGYGSK-RIGGDMGWLESAL-NEYVAAVRKVAVRVLEAMAE-GLAGGDQVFRVNHYPPC
-P-TGFGEHTDPQLISVLR-SNTSGLQIQWVSVPSRDA--FFVNVGDSLQVLTNGRFKSV
KHRV-VT-KSRVSFIYFAGPALEQRIVPLPYKEFTWEYKKA
>LOC_Os01g55240.1
VPVVDLGSAARA-VVDACERYGFFKVVN--HGVATD--TMDKAESEAVRFF-SQTQPDKD
RSGYGSK-RIGGDMGWLEAAL-NEYISGVRKVAVRVMEAMSE-GLEGSDQVFRVNHYPPC
-R-TGFGEHTDPQLVSVLR-SNTSGLQIQWVSVPSRDS--FFVNVGDSLQVLTNGRFKSV
KHRV-VA-KSRVSFIYFGGPPLAQRIAPLPYKEFTWEYKKA
>KQK09810
MPVIDLCSAPRA-IADACERFGFFKLVN--HGVATD--AMDRLESEAVTFF-SQPQADKD
RSGYGSK-RIGGDMGWLERAL-NEYIGAVRKVAVRVMEAMAE-GLAGSDQVFRVNHYPPC
-A-TGFGEHTDPQLVSVLR-SNTSGLQIQWVSVPSRDA--LFVNVGDSLQVLTNGRFKSV
KHRV-VA-KSRVSLIYFGGPPLTQRIAPLPYTEFTWEYKKA
>HORVU3Hr1G072810.1
MPVVDLSRAPRA-IADACERFGFFKLVN--HGVALD--AMDRLESEAVRFF-SLPQADKD
RSGYGSK-RIGGDMGWLEAAL-NEYIAAVRKVAVRVMEAMAE-GLEGSDQVFRVNHYPPC
-H-TGFGEHTDPQLISVLR-SNTSGLQIHWVSVPSRDA--FFVNVGDSLQVLTNGRFKSV
KHRV-VA-KSRVSMIYFGGPALTQRIAPLPYKDFTWEYKKA
>LOC_Os05g43880.1
LPVVDLAAAAGE-VVRACERFGFFKVVS--HGVGEG--VVGRLEAEAVRFF-ASPQAAKD
AHGYGSK-RIGGDMGWLEDAA-NKYVGAMRGMARTVLEMVAE-GLAASDQILRLNHYPPC
PP-TGFGEHTDPQLISILH-SNTSGLQVQWVTVPPPAS--FLVIVGDSLQVMTNGRMRSV
RHRV-VA-KSRVSMIYFGGPPLEQRIAPLRYEEFTWEYKKA
>Zm00001d038695_P001
VPAVDLSSAALA-VVDACERFGFFKVVN--HGVPAG--VVDRLEAEAVRFF-ASPQAAKD
ACGYGNR-RIGGDMGWLEDAV-NQYVASVRGLATSVLEAVAE-GLAASDQVFRINHYPAC
-P-TGFGEHTDPQLVSVLR-SNTPGLQLRWVPVPPRDA--FFVIVGDSLQVLTNGRLKSV
RHRV-VA-KPRVSMIYFAGPAPAQRIAPLPYRDFTWDYKKA
>EES19801
VPAVDLSSAALA-VVDACERFGFFKVVN--HGVPTG--VVDRLEAEAVRFF-ASPQADKD
ACGYGNK-RIGGDMGWLEDAV-NQYVAAVRGLATSVLEAVAE-GLAASDQVFRINHYPAC
-P-TGFGEHTDPQLVSVLR-SNTAGLQVRWVPVPPRDA--FFVIVGDSLQVLTNGRLKSV
RHRV-VA-KPRVSMIYFAGPAPAQRIAPLPYRDFTWDYKKA
>KQK05406
VPAVDLSAAADD-VVRACERFGFFSVVN--HGVARG--VVERLEHEAALFF-SWPQADKD
ASGYGSK-RIGGDMGWLEDAI-NEYVGAMRGLARTVLEMVAE-GLEASDQVFRLNHYPPC
-P-TGFGEHTDPQLVSILH-SNTAGLQIKWVSVPPRDA--FFVNVGDSLQVLTNGRLKSV
RHRV-VARKSRVSMIYFGGPPVAQRIAPLPYREFTWEYKKA
>HORVU1Hr1G076730.2
VPAVDLSSAAAD-VVRACERFGFFSVVN--HGVPAG--VVDRLEAEAVRFF-ASTQAEKD
ASGYGSK-RIGGDMGWVEEAI-NAYVSAMRGLARTVLEMVAE-GLEASDQVFRVNHYPPC
-P-TGFGEHTDPQLVSILH-SNTAGLQVRWVSVPPRDA--FFVNVGDSLQVLTNGRLRSV

RHRV-VALKSRVSMIYFAGPPLAQRIAPLQYRDFTWEYKKA
>HORVU1Hr1G086710.2
VPSVDLSAAGAA-VAAACRRVGFFRATN--HGVPAA--LTDALEARAEAFF-ALPHEDKL
EAGYGSK-SIGGDVGWLEAAL-EEYTDAVREVGARVLELMAD-GLDGADEMVRVNHYPPC
-P-TGFGEHTDPQIISVLR-SNTGGLQIRWVPVAPPDS--LFVNVGDSLQVLTNGRFRSV
KHRV-VA-QPRLSVIYFGGPAPTQRIAPLPYRDFTWEYKKA
>HORVU1Hr1G086810.2
VPSVDLSAAAAA-VADACRAVGFFRATN--HGVPAA--LTDALEERAASFF-ALPHKDKM
DAGYGSK-NIGGDVGWLE----EEYTDAVREVGARVLELMAD-GLGGADEMVRVNHYPPC
-P-TGFGEHTDPQIISVLR-SNTAGFQIRWVPVAPTDS--LFVNVGDSLQVLTNGRFRSV
KHRV-VA-QPRLSVIYFGGPAPTQRIAPLPYRDFTWEYKKA
>KQK04908
IPTVDLSSAARA-VAEACRGVGFFRATN--HGVPSS--LAATLEADAMAFF-ALPDKDKQ
STGYGSR-RIGGDVGWLELAL-HEYTRAVRELSGRVLELMAE-GLGSEEELVRVNHYPPP
-T-TGFGEHTDPQLISLLR-SNTAGYQIRWVNVAPPDS--FFVNVGDTLQVLTNGRFRSV
KHRVLVAKASRLSVIYFGGPAPAQRIAPLPYRDFTWEYKAA
>KQK04914
IPTVDLSSAARA-VADACRGVGFFRATN--HGIPSS--LAAALEARAMAFF-ALPHEDKV
DAGYGSR-SIGGDVGWLEAAL-EEYTGAVRNASGKVLELMAE-GLGSEEELVRVNHYPPT
-K-TGFGEHTDPQIISLLR-SNTAGLQIPWVNVAPPDS--LFVNVGDCLQVLTNGRFRSV
KHRV-VA-ASRLSVIYFGGPAPAQRIAPLPYRDFTWEYKAA
>LOC_Os05g48700.1
IPCIDLSAAAAA-VADACRTLGFFKATN--HGVPAG--LADALESSAMAFF-ALPHQEKL
DMGYGSK-SIGGDVGWLEAAV-EAYTGAVRGVGCRVMELMAE-GLEGSDEMLRVNHYPPC
-L-TGFGEHTDPQIISVLR-SNTAGLQIRWVPVPPPDS--FFVNVGDSLQVLTNGRFRSV
KHRV-LAEESRLSVIYFGGPAASQRIAPLEYREFTWEYKKA
>KQL14341
IPCVDLSAAAAA-VADACRSVGFFRATN--HGVPAS--VADALEAGAMAFF-ALPAQDKV
DMGYGSK-SIGGDVGWLE----EEYTAAVREVGGRVLELIAE-GLEGGDELVRVNHYPPC
-P-TGFGEHTDPQIISVLR-SNTAGLQIRWVPVPPPES--FFVNVGDSLQVLTNGRFRSV
KHRV-VASHSRLSVIYFGGPAPSQRIAPLPYREFTWEYKRA
>Zm00001d038996_P001
VPCVDLSAAGAA-VADACRSVGFFRATN--HGVPAR--VADALEARAMAFF-ALPAQEKL
DMGYGSK-SIGGDVGWLE----EEYTAALREVCGRVLELIAE-GLEGSDEVVRVNHYPPC
-P-TGFGEHTDPQIISVLW-SNTAGLQIRWVPVPPPES--LFVNVGDSLQVLTNGRLRSV
KHRV-VA-RSRLSVIYFGGPAPSQRIAPLPYREFTWEYKRA
>EES19940
IPCVDLSAAAAA-VADACRGVGFFRATN--HGVPAR--VVEALEARAMAFF-ALPAQEKL
DMGYGSK-RIGGDVGWLE----EEYTAAVREVCGRVLELIAE-GLEGSDELVRVNHYPPC
-P-TGFGEHTDPQIISVLR-SNTAGLQIRWVPVPPPES--FFVNVGDALQVLTNGRFKSV
KHRV-VAAQSRLSVIYFGGPAPSQRIAPLPYREFTWEYKTA
