# Supplementary material for: Genome-Wide Identification and Evolution Analysis of the Gibberellin Oxidase Gene Family in Six Gramineae Crops
Source: Genes (Basel). 2022 May 12;13(5):863. doi: 10.3390/genes13050863 (PMC9141362; doi:10.3390/genes13050863)
Supplement: Supplementary file 1 [file genes-13-00863-s001.zip › Supplementary file 2.pdf]

>KQJ85786

--MEQRRAESPI
PADGAAA--SKLEEL-----------------------------------ELPTLDLEEE
S------------------------------GLTEALAAACRDLGVFRLAN--HGI----
PAD--LSDRLFSLTRHLL-EEPDAEKKAK-------------LPG-----YFSGTP-ALS
LRV--KELNWVEG------FHV----------------APAAADADPDPT----------
--AADDDIGGEFGSFR-------------------------------EVT-AEYVSRMAR
IARKLFDAMDL-GL----D-------------AS------QRASYL-----AEHDGTFRA
YRYPAC-PAG-----------------------------GH--IGMEAHTDSSVLSILNQ
QD---LQESSLQVLLRPQ---------G-------TWRSVQP----VEGT--LVVNIGDM
MQAITGDAYR-----SVEHRV-VP------L--PD-TDRMSLCYFAFPQDDAVISGCG--
---ED-------DGE----KGSC---YRPFSY-------REFREQVQADIK---------
-------------------------------ATGAK------------------VGLARF
RRGASAPELASAIGTEAGL-------------------------------------
>HORVU3Hr1G117870.1

--MDRQPVEC
----------GLEEV-----------------------------------ELRAVDLEAE
E--S---------------------------ILTEELAAACRDPGVFRLVN--HGV----
PSN--LTARLLALARGLL-ELDAATKSR--------------LPG-----YFCGTP-ALA
ALPV-KEPNWLEG------LHV------------------EAAGDTCLCS----------
--PDGGSAVAEF----------------------------------MEAVSGEYVAHMAR
IARKVFDTLASGEL----G---L----D----SE------QRATYL-----TERGCIFRA
YRYPAA-AAS-----------------------------GQ--VGMEAHTDSSVLSILN-
QD---T-VGGLQVL--YR---------G-------CWRAVRP----VEGA--LVVNVGDM
LQAMSGDAYR-----SPEHRV-VA------P--AG-ADRMSLCYFAFPEEEAVIVGGRWR
GRSSS-------GGE----Q-RETRRYQGFSY-------REFREQVQADVK---------
-------------------------------ATGSK------------------VGLARF
RVAVAQS---
>LOC_Os04g33360.1

--------------------------MGWASAAATCGRLQPGGGGGESSSTTTTRTTQMD
PSCPPFP---QPEAP----PLPLQLQLQPGLPR----------------LELPTLDLERV
G--G---------------------------EDRAALVAACRDLGAFRVVN--HGV----
PGE--LRRRLLELGKQLLGRDTFELKKA--------------RPG-----YFWGTAALKS
LRV--KEVNWLEG------LHV--------------DLVPGSSSSSSQVG----------
--DGDDDDDDGWMRIR-------------------------------ALM-AEYGDHMAR
IARKLFDALAA-EL----G---L----D----HH------QAASYL-----AERQGFLRL
YRYPPC-PSS---------------------------A-SC--LGMEPHTDSSVLSIILG
QD---H-VGGLQVI--RD---------G-------AWRDVAP----APGE--LLVNLGDM
MTAISGGSYQ-----SVRHRV-LA------S--RPSTERVSCCYFAFPQEDAVVEAPS--
----G-------IGG------GV---YRPFSY-------REFREQVQADIK---------
-------------------------------AVGTK------------------VGLSRF

YATATRYTR---
>KQL09066

--MAVESSETPC
PPFPLIGDEDDVGGHENQAAAEVPQGEA---------------------LELPTVDLEAP
G------------------------------PA---LDAACRGLGIFRLAN--HGV----
PAD--LTARLFALARDLLGRTPFPEKKA--------------QPG-----YFWGTPEEPS
RRVRPRDVNWIEG------YHV----------------HLAQPRPIAVGP----------
--PPPSDDDPAL----------------------------------GDLV-AEYADHMAR
VARRLFDAIAE-AL----C---L----D----AD------RTASYI-----NEHGGYLRV
HRYPRC-PEP-----------------------------GH--HGIQAHTDSTTLSIIN-
QDAASGGSGGLQVH--HD---------G-------AWRDLAAPGVPDDGA--LLVNLGDM
AKAISADAYR-----SVPHRV-VA------S--MGDDERLSLCYFAFPRDDAVVS-----
------------CDG------SR---YRPFTV-------PEFRAQVQADLK---------
-------------------------------AIGSK------------------VGLERF
LRH---
>KQL09067

--MAAAMADPSC
PPFPLLAEEGHHEQH----EHQEVPHGG--------------------ELEVPTVDLQAP
G--EA-------------------------------LAAACRRLGVFRLAN--HGV----
PGD--LSARLFALARDLLGRAPFLDKQA--------------QPG-----YFWGTP-ALS
SLRV-RDVNWVEG------FHV-ALAGQQ----------QHRPVTAAAAP----------
--PPSDDLAAAL-R---------------------------------DLA-REYGAHMAR
VARALFDALAA-AL----G---L----G----SE------QSSAYL-----AERDGFLRV
YRYPPC-PEP-----------------------------GH--LGMEAHTDSSVLSVIN-
QD---L-VGGLQVL--HD---------G-------AWRDVAPAGRGDAGT--LLVNLGDM
ARAISGDAWR-----SVRHRV-AA------S--RA-AERLSLCYFAFPRDDAVITC----
------------AGG------SR---YRPFTY-------AEFREQVQADIK---------
-------------------------------ATGSK------------------VGLERF
LRH---
>Zm00001d035994_P001

--MASGSDSRTAAD
PSYPPFPLLEQDDEG----GGHVHEQEQEARRG--------------GALELPTVDLEAP
G--EA-------------------------------LAAACRELGVFRLAN--HGV----
PAA--LSARLFALARAALAGAPFRDKRA--------------QPG-----YFWGTP-ALS
LRV--RDVNWVEG------FHV---------------ALGEAQQQQQHAP----------
--VPPPPLGDLL-----------------------------------GLARAEYGAHMTR
VARTLFDALAP-AL----G---L----G----AA------QAASYL-----AERDGFLRV
YRYPRC-PEP-----------------------------GR--LGMEAHTDSSVLSVIN-
QE---EGAGGLQVL--HG---------G-------AWRDVAP----AAGASTLLVNLGDM
ARAISGDAYR-----SVRHRV-AA------S--QA-AERLSLCYFAFPRDDAVIT-----
------------CGA------GR---YRPFTY-------AEFREQVQADIK---------

-------------------------------ATGSK------------------VGLERF
LLHH--
>OQU85872

--MALGSESQAATTAD
PSCPPFP---LLEDD------GGHIHEQEQEVP--------SGA----ELELPTVDLDAP
G--EA-------------------------------LAAACRGLGVFRLAN--HGV----
PAS--LSARLFATARAALAGAPFHDKRA--------------QPG-----YFWGTP-ALS
LRV--RDVNWVEG------FHV--------------------ALGGHQQP----------
--APGPPLGDLL-----------------------------------DLA-AEYGAHMAR
VARALFDALAA-PL----G---L----G----AD------QAATYL-----AERDGFLRV
YRYPRC-PEP-----------------------------GH--LGMEAHTDSSVLSVIN-
QD---A-VGGLQVF--HD---------G-------AWRDVAP-GAGETGT--LLVNLGDM
ARAISGDAYC-----SVRHRV-AA------S--QA-AERLSLCYFAFPQDDAVIS-----
------------CAG------GR---YRPFTY-------AEFREQVQADIK---------
-------------------------------ATGSKKPVGAPLAGPHGTVTVSIAGANAF
NMVLSWTV--
>KQK21235

--MEDHEYEQDESNPPLL
ATYKHLLDGEHRRLP----LGVVAGDEE------------------DDQCDLPVIDLAPL
L--LDLESDQQTRNNGGSSSA----------ACRAAMVRAASEWGFFQVTN--HGV----
PQP--LLDELHGQQLRAF-RRPFHRKLAPGDGDDEDQLLL--SPE----AYRWGNP-TAT
-CL--HQLSWSEA------YHV-----------------------PITTP----------
--SPETKTTTAT-R---------------------------------RVI-HEVSAAMSK
LARRILSVLAA-EH----G-------------PP------PET--------ETTTCFLRL
NRYPQA-PGSGGGHGH-----------------------GV--LGLCGHTDSDFLTILR-
QD---DHVGGLQLL--LD--------DK---DGGRRWRTVRP----NPGA--LTVNVGDL
LQAWTNDVYA-----SVEHRV-VA------R--PD-RERFSVAFFLCPSYDTLIRPPL--
---------PGKTTT----S-QV---YRNFTF-------GEYRSQVREDVR---------
-------------------------------LTGRK------------------VGLPRF
RRHVSPSPASLTHQLS--
>LOC_Os07g01340.1

---MEEHDYDSNSNPPLM
STYKHLFVEQHRLDM----DMGAIDVDE---------------------CELPVIDLAGL
M--EAEQ------------------------VCRADMVRAASEWGFFQVTN--HGV----
PQA--LLRELHDAQVAVF-RRPFQEKVTERLLGF--------SPE----SYRWGTP-TAK
-CL--EQLSWSEA------YHI--------------PMTTPRPSTSIRAR----------
---AVI-EEVSRAMYE
LAQKLAEILMR-GL---PG-------------AG------EGETMV---TTREETCFLRL
NRYPPC-AMA---------------------------M-GG--FGLCPHTDSDLLTIVHQ
QQ---DTVGGLQLL--KG---------G-------RWVAVKP----SPST--LIVNVGDL
LQAWSNDVYK-----SVEHRV-MA------N--AT-LERFSMAFFLCPSYHTLIIPSS--

-SHVH-------DDD------AH---YRSFTF-------GEYRKQIMEDVR---------
-------------------------------STGRK------------------IGLHRF
RTR---
>KQK85900

--MDDESNPPLM
TTYKHLL---DTHPHRRLDAVDDHNSGC----------------------VLPVIDLGSL
QHAPE--------------------------QCRAAIVRAASEWGFFQVTN--HGV----
PQP--LLDELHDAQVAVF-RRPFERKLREPLLDF--------SPE----SYRWGTP-TAT
-CL--EQLSWSEA------YHI-------------------PMITPAAAA----------
--AAGDDDRTRL------------------------------------VI-EEVSTAMSK
LALQLAGILVA-DL----R-------------GD------EEKDMV--ARCTRNTCFLRL
NRYPAC-GAA---------------------------T-GA--FGLCPHTDSDFLTILH-
QD---A-VGGLQLL--KA---------G-------RWVAVKP----NPGA--LIVNVGDL
LQAWSNDRYR-----SVEHRV-MA------S--AA-SERFSVAFFLCPAYDTLIRPRSTT
SCSGG-------GAS----P-PR---YRSFTF-------GEYRNQIKEDVR---------
-------------------------------LTGRK------------------IGLQRF
RLQQQLQGGGPL--
>Zm00001d018617_P001

--MAMEEDDDYEPPLM
ATYKHLL---DSHPH------RQLGAGAAQDDE--------------DCFLLPVIDLSSL
L--------------PPVHHRKQSSSAAAAEQCRASIVRAASEWGFFQVTN--HGV----
PQV--LLDELHQAQAGVF-RRPFQLKAHQPLLDF--------SPE----SYRWGTP-TAT
-CL--EQLSWSEA------YHI--------------PTTTTTTGNDDKTR----------
---LVV-EEVSTAMSK
LAQRLAGILVA-EL----G--------EDS--AA------AVVSRC-----TRSTCFLRL
NRYPPC-AAA---------------------------A-SGVVYGLCPHSDSDFLTILH-
QD---G-VGGLQLV--KD---------G-------QWVAVKP----SPGA--LIVNVGDL
LQAWSNDRYR-----SVEHRV-MA------S--AT-RERFSAAFFLCPSYDTLIRPRC--
----G-------AGG----PPPR---YESFTF-------GEYRNQIREDVR---------
-------------------------------LTGRK------------------LGLQRF
RKAE--
>EER95722

---MEDYDYEPPLM
ATYRHLL---DSHPH------RLDVVDHRSGAD-----------DDEEGFLLPVIDLSSL
L--EQS---------SSGAEA------AAEQCRAS-IVRAASEWGFFQVTN--HGV----
PQA--LLDELHQAQVAVF-RRPFHLKASQP--------LLDFSPE----SYRWGTP-TAT
-CL--DQLSWSEA------YHI-------------------------PTT----------
--NTTAAADDKT-R---------------------------------LVV-EEVSTAMSK
LAQRLAGILVA-DL-LL-GDSSI----GD---GEDDDTAAAVVSRC-----TRSTCFLRL
NRYPPC-PAP---------------------------S-GA--YGLCPHTDSDFLTILH-
QD---G-VGGLQLV--KA---------G-------RWVAVKP----NPGA--LIVNVGDL

LQAWSNDRYR-----SVEHRV-MA---------SDARERFSVAFFLCPSYDTLVRPRC--
----G-------AGG----P-PR---YESFTF-------GEYRNQIREDVR---------
-------------------------------LTGRK------------------LGLQRF
RKPE---
>HORVU2Hr1G090030.9

--XXXXXXXXXXXXXXXXXXXX
XXXXXXXXXXXXXXX-----------------------------------XXXXXXXXXX
X------------------------------XXXXXXXXXXXXXXXXXXXX--XXX----
XXX--XXXXXXXXQARLF-RLPFDTKEKAGL-----------LNG----SYRWGNP-TAT
-SL--RHLSWSEA------FHV-------------------PLATISRED----------
--CDYGKLSSL--R---------------------------------YVLTSXXXXXXXX
XXXXXXXXXXX-XX----X---X---------XX------XXXXXX-----XXXXXXLRL
NRYPAC-PFA---------------------------P-DT--FGLVPHTDSDFLTILC-
QD---Q-VGGLQLM--KD---------A-------RWVAVKP----HPDA--LIVNVGDL
FQAWSNNRYK-----SVEHKV-VA------N--YK-AERFSVAYFLCPSYDSPVGTCS--
------------EPS----P------YRPFTF-------GEYRRKVQDDVK---------
-------------------------------RTGKK------------------IGLPNF
LK---
>LOC_Os02g41954.1

--MPAIADCAADPPLA
DSYYTLLRLGGDDDD------DACTKVTTTPQP-------------VSECELPMIDVGCL
T--APTGAA------AAAAVGQQHQAEERAACAAA-IAAAAAEWGFFQVVN--HGV----
AQE--LLEAMRREQARLF-RLPFEAKSSA-------------GLLND--SYRWGTP-TAT
-SL--RQLSWSEA------FHL-------------------PLAGISGKS----------
--CNYGDLTSL--R---------------------------------DVT-REVADAMSR
LARALARVLAE-SL--------LGHAAGE-----------RFPEGC-----DDATCFLRL
NRYPPC-PFP---------------------------PDDA--FGLVPHTDSDFLTVLC-
QD---H-VGGLQLM--KG---------S-------RWVAVKP----IPGA--LIVNIGDL
FQAWSNNRYK-----SVEHRV-MT------N--AT-TERYSVAYFLCPSYDSPIGTCR--
------------EPS----P------YKAFTF-------GEYRRRVQEDVK---------
-------------------------------KTGKK------------------TGLSNF
LV---
>KQK00433

--MPAFAESAAEPPLA
DSYHALLRRAGADGR----NKDAHCTPTVHADH-----VLPAPDMAVSECELPMIDVGCL
T--TGGGS-------AEERAA----------CAAA-IAGAAEDWGFFQVVN--HGV----
KQE--LLEAMRREQTRLF-RLPFEAKATA-------------GLLNH--SYRWGTP-TAT
-SP--AQLSWSEA------FHV------------------PLAAVSGDPP----------
--CNYGQLTTL--R---------------------------------DVT-QEVATAMSK
LANTLARVLAE-RL----G--------HA---GE------RFPEGC-----DERTCFLRL
NRYPPC-PLS---------------------------P-DA--FGLVPHTDSDFLTVLC-

QD---Q-VGGLQLM--KG---------S-------RWLAVKP----IPNA--LIVNIGDL
FQAWSNNRYR-----SVEHKV-VT------N--AT-TERYSVAYFLCPSYDSPIGACE--
------------EPS----P------YRTFTF-------GEYRRRVQEDVK---------
-------------------------------KTGKK------------------VGLPGF
LL--
>KQL30590

--MPAFAGSAAEPPLA
DSYHALL---RRHGG------GDNDDGAHTAAV------------PVAECELPMIDVGCL
T--RD----------GGGSEA------ERAACAAA-IARAAEEWGFFQVRN--HGV----
APG--LLDAMRREQARLF-RLPFEAKSTA-------------GLLND--SYRWGTP-TAT
-SP--RQLSWSEA------FHV-------------------PLAGVSGDR----------
--CNFGDLTA--LR---------------------------------DVT-REVAGAMSK
LAGTLARVLAE-AL--------L---------AGRRPAGERFPEGC-----DETTCFLRL
NRYPPC-PVS---------------------------P-DA--FGLVPHTDSDFLTVLC-
QD---H-VGGLQLM--KG---------A-------RWVAVKP----IPGA--LIVNIGDL
FQAWSNNRYK-----SVEHKV-VT------N--AT-TERYSVAYFLCPSYDSPIGTCE--
------------EPS----P------YRTFTF-------GEYRSKVQEDVK---------
-------------------------------RTGKK------------------IGLPNF
LV--
>Zm00001d017294_P001

--MPAFAGGAAEPPLA
DSYYALLRRGNDDEGAYTTSTAPWDDVS----------------LPVAECELPMIDVGCL
TTADDGSSPEAEAERA---------------ACAAAIARAAEEWGFFQVRN--HGV----
PQE--LLEEMRREQARLF-RLPFETKATAGL-----------LND----SYRWGTP-TAT
-SP--RQLSWSEA------FHV-----------------PLAGVSGSGTT----------
--CDFGDLTTLR-----------------------------------DVT-REVAGAMSK
LAGTLARVLAE-AL--------L----GRRPAGE------RFPEGC-----DETTCFLRL
NRYPAC-PIS---------------------------P-GA--LGLVPHTDSDFLTVLC-
QD---QQVGGLQLM---K---------G------DSWVAVKP----IPGT--LVVNIGDL
FQAWSNNRYK-----SVEHKV-MT------N--AR-TERYSVAYFLCPSYDSPIGT----
------------CEE----P-SL---YRTFTF-------GEYRRKVQEDVK---------
-------------------------------RTGKK------------------VGLPNF
LAQT--
>KXG30679

--MPAFAGSAAEPPLA
DSYYALLRRRGGGGN-----NDDEEASGGAYYYDTTSSTVPSSDDDVAECELPMIDVGCL
T--------------TLTRSEHGSSSEAERAACTAAIARAAEEWGFFQVRN--HGV----
SQE--LLDEMRREQARLF-RLPFEAKATAGL-----------LNH----SYRWGTP-TAT
-SP--QQLSWSEA------FHV---------------PLAGVSGSAAAGT----------
--CDFGDLTTL--R---------------------------------DVT-REVAGAMSK
LAGTLARVLAE-EARPRPC---LPAGGGGGGGE------RFPEGC-----DETTCFLRL

NRYPPC-PIA---------------------------A-DA--FGLVPHTDSDFLTVLC-
QD---QQVGGLQLM--KG---------G-------RWVAVKP----IPGA--LIVNIGDL
FQAWSNNRYK-----SVEHKV-MT------N--AK-TERYSVAYFLCPSYDSPIGT----
------------CEE----P-SL---YRTFTF-------GEYRRKVQEDVK---------
-------------------------------RTGKK------------------VGLPNF
LV--
>HORVU4Hr1G072540.2

--YSSFLFRFRAPPIL
ASRD------ARESK---
-------------------------------ACADAMARAASEWGFFQVVN--HGV----
GLE--LLEEMRREQAKLF-RLPFGTKDKAGL-----------LNG----SYRWGNP-TAT
-SL--GQLSWSES------FHV-------------------PLPSVSPEG----------
--CDYGKLSTL--R---------------------------------GVM-QEVADAMSR
VADTVAGTLAE-NL----G-----HEAGGGGGES------AFPGGC-----DGTTCFLRL
NRYPAC-PFA---------------------------A-DS--LGMVPHTDSDFLTILF-
QD---Q-VGGLQLI--KD---------A-------RWVAVKP----HAEA--LIVNVGDL
FQAWSNNKYK-----SVEHKV-VA------N--SS-AERFSVAYFLCPSWDSPVGT----
------------CGE----P-SP---YKPFTF-------GEYRRSVQDDVE---------
-------------------------------RTGKK------------------IGLPNF
LKRSTVDVTEP---
>KQK98136

--MPAFTESRAAAEPPLA
ESYLDLLRRGGGGIA------PPGEGRV-----------------AVQERELPLIDLGCL
M--------------TTSGQQGSRSAREARAACAYAMARAASEWGFFQVTG--HGV----
GRA--LLERLRAEQARLF-RLPFETKARAGL-----------LNG----SYRWGAP-TAT
-SL--RHLSWSEA------FHV-------------------PLASISGSG----------
--CDFGELGSL--R---------------------------------GVM-QEVADAMSR
VAKTVAVALAG-SLLQ--G--------HHAAAAA------AFPAGC-----DETTCFLRL
NRYPAC-PFA---------------------------P-DT--FGLVPHTDSDFLTVLC-
QD---Q-VGGLQLM--KD---------A-------RWVAVKP----HPDA--LIVNIGDL
FQAWSNNRYK-----SVEHKV-VA------N--AK-AERFSAAYFLCPSYDSPVGT----
------------CGE----P-SP---YRSFTF-------GEYRRMVQEDVK---------
-------------------------------RTGRK------------------IGLPSF
LKQQQPQ---
>Zm00001d002999_P001

------------------------------------MRYVAATPTMPSLVAESAAEPPLV
DSYLELLRRGGGGGG----IAAATEGCV-------------------QERELPLIDLTCL
Q--GSAG--------EAARTT----------CADA-MARAASEWGFFQVTG--HGV----
SRA--LLERLRAEQARLF-RLPFETKAKAGL-----------LNG----SYRWGAP-TAT
-SL--RHLSWSEA------FHV---------PLASIS----------GTA----------
--CDFGELSS--LR---------------------------------DVV-QEVADAMSR

VAKTVAVALAGSLL----G---H----DE---AA------AFPAGC-----GETTCYLRL
NRYPAC-PFA---------------------------A-NT--FGLVPHTDSDFLTVLS-
QD---Q-VGGLQLM--TD---------A-------GWVAVKP----RPDA--LIVNIGDL
FQAWSNNLYK-----SVEHKV-VA------N--AA-AERFSAAYFLCPSYDSLVGT----
------------CGE----P-SP---YRDFTF-------GEYRRKVQEDVK---------
------------------------------RTGRK------------------IGLPNF
LKHRPPPQSRPA---
>KXG26736

--------------------------------MRYVAATPTMPALTTTTAESAAEPPLA
DSYLDLLRRGGIATP------PRTEGRCCVQER-----------------ELPLIDLSCL
Q--QRSGGGG-----GSARAA----------CADA-MARAASEWGFFQVTG--HGV----
SRA--LLERLRAEQARLF-RLPFETKAKAGL-----------LNG----SYRWGAP-TAA
TSL--RHLSWSEA------FHV-------------------PLASISGAA----------
--CDFGDLSSL--R---------------------------------GVM-QEVADAMSR
VAKTVAVALAG-SLLQGGG---H----DEHEAAA------AFPAGC-----DETTCYLRL
NRYPAC-PFA---------------------------A-DT--FGLVPHTDSDFLTVLC-
QD---H-VGGLQLL--KD---------G-------RWVAVKP----RPDA--LIVNIGDL
FQAWSNNRYK-----SVEHKV-VA------N--AT-AERFSAAYFLCPSYDSPVGT----
------------CGE----P-SP---YRDFTF-------GEYRRKVQEDVK---------
------------------------------RTGRK------------------IGLPNF
LKQQSRQ---
>LOC_Os04g44150.1

---MPAFADIAIDPPLA
DSYRALALLRRDRDG----GIAPPAVQMVGSGG------------AVLERDLPMVDLERL
T--RG----------GAGERK----------ACAGAMARAASEWGFFQLTN--HGV----
GRE--LMEEMRREQARLF-RLPFETKEKAGL-----------LNG----SYRWGNP-TAT
-SL--RHLSWSEA------FHV-------------------PLASISGAD----------
--CDFGDLTSL--R---------------------------------GVM-QEVAEAMSR
VANTVAAALAE-ELTGRGG--------GGASAAP------WFPAGC-----DETTCFLRL
NRYPAC-PFA---------------------------A-DT--FGLVPHTDSDFLTVLC-
QD---Q-VGGLHLM--KD---------S-------RWVAVRP----RPDA--LVVNIGDL
FQAWSNNRYK-----SVEHKV-VA------N--AK-TDRLSVAYFLCPSYDSLVGT----
------------CGE----P-SP---YRAFTF-------GEYRKKVQEDVR---------
------------------------------TTGKK------------------IGLPNF
FKHSSVQ--
>KQJ83649

---MPALFADGATSDPPLA
DSYRALLRSGGIAHP------PEESLSP------------------VLERDLPMIDIECL
I--T-----------TGGEG-GASSMRKKKACADA-MARAASEWGFFQVTN--HGV----
GKE--LLEEMRKEQARLF-RLPFETKRKAGL-----------LNG----SYRWGNP-TAT
-SL--RQLSWSEA------FHV---------PLASIS----------GRD----------

--CDYGKLSS--LR---------------------------------GVM-QEVADAMSR
VADAVAGALAE-KLGQEAG-------------SA------LFPAGC-----DGTTCFLRL
NRYPAC-PFA---------------------------P-DT--FGLVPHTDSDFLTILC-
QD---Q-VGGLQLM--KD---------S-------RWVAVKP----HPDA--LIVNIGDL
FQAWSNNRYK-----SVEHKV-VA------N--AK-AERFSVAYFLCPSYDAPVGT----
------------CGE----P-SP---YRSFTF-------GEYRRKVQDDVK---------
------------------------------RTGKK-------------------IGLPNF
LKHPPIVDGLK---
>HORVU2Hr1G118350.1

--------------------------------------MPSQLNKDPHNRYFDLGAAREV
PDTHAWD---GQHEL------PVVDGGVGAGDD-----------------AVPVVDMRDP
R------------------------------AAEA-VARASEQWGAFLLEG--HGV----
PTE--LLARVEAGIAGMF-ALPTPEKMRAAR-----------HDG-D--LYGYGPP-LIA
SYV--SKNMWSEG------YTL------------------TPANLHAEFR----------
--KIWPDAGQHY-RHFS------------------------------GVM-EEFLKEMRS
LANRLMELFLV-AL----G---L--TAEQIAGVE------AEHNLT-----ESMTETMHL
NWYPKC-PDP---------------------------T-RA--LGLKGHTDSGFFTFVM-
QS---Q-VPGLHLF--RH---------G---PPADRWVEVPA----VPGA--LIVNIGDL
FQILTNGRFR-----SVYHRV-VV------N--RE-RERISVAYFLIPPADVKVAPLK--
-EVVG-------GGK------PV---YRALTWSESIVVRKEAFANHGADLE---------
------------------------------FGKGR-------------------TALDML
SISSDEDDGAEHHRD---
>HORVU3Hr1G022840.8

--------------------------------------MPTPSHLSKDPHYFDFRAARRV
PETHAWP---GLHDH------PVVDGGGAGGGP-------D---------AVPVVDMRDP
C------------------------------AAEA-VALAAQDWGAFLLQG--HGV----
PLE--LLARVEAAIAGMF-ALPASEKMRAVR-----------RPG-D--SCGYGSP-PIS
SFF--SKCMWSEG------YTF------------------SPANLRSDLR----------
--KLWPKAGHDYRHFC-------------------------------AVM-EEFHREMRV
LADKLLELFLV-AL----G---L--TGEQVAAVE------SEHKIA-----ETMTATMHL
NWYPKC-PDP---------------------------K-RA--LGLIAHTDSGFFTFVL-
QS---L-VPGLQLF--RH---------G-----PDRWVTVPA----VPGA--MVVNVGDL
FHILTNGRFH-----SVYHRA-VV------N--RD-SDRISLGYFLGPPAHVKVAPLR--
-EAL--------AGT----P-AA---YRAVTW----PEYMGVRKK-AFTTG---------
------------------------------ASALK---------------------MVAI
STDDAADIDSGAGCVQPGSRER----------------------------------
>LOC_Os01g08220.1

---------------------------------------MPTPSHLKNPLCFDFRAARRV
PETHAWP---GLDDH------PVVDGGGGGGED-----------------AVPVVDVGAG
D------------------------------AAAR-VARAAEQWGAFLLVG--HGV----
PAA--LLSRVEERVARVF-SLPASEKMRAVR-----------GPG-E--PCGYGSP-PIS

SFF--SKLMWSEG------YTF------------------SPSSLRSELR----------
--RLWPKSGDDY-------------------------------LLFCDVM-EEFHKEMRR
LADELLRLFLR-AL----G---L--TGEEVAGVE------AERRIG-----ERMTATVHL
NWYPRC-PEP---------------------------R-RA--LGLIAHTDSGFFTFVL-
QS---L-VPGLQLF--RR---------G-----PDRWVAVPA----VAGA--FVVNVGDL
FHILTNGRFH-----SVYHRA-VV------N--RD-RDRVSLGYFLGPPPDAEVAPLP--
-EAVP-------AGR----S-PA---YRAVTW----PEYMAVRKK-AFATG---------
---------------------------------GSA--------------------LKMV
STDAAAAADEHDDVAAAADVHA----------------------------------
>KQL04660

---------------------------------------MPTPSHLKNPLYFDFRAARRV
PESHAWP---GLDDH------PVVDGGGAPGSP----------------DAVPVVDLREP
G--A---------------------------AAVARVARAAEQWGAFLLTG--HGV----
PAE--LLARVEDRVACMF-ALPAADKMRAVR-----------GPG-D--ACGYGSP-PIS
SFF--SKCMWSEG------YTF------------------SPASLRRDLR----------
--KLWPKAGDDY-DSF------------------------------CDVM-EEFHKEMRA
LADRLLELFLR-AL----G---L--TGEQVGAVE------AERRIG-----ETMTATMHL
NWYPRC-PDP---------------------------R-RA--LGLIAHTDSGFFTFVL-
QS---L-VPGLQLF--RH---------G-----PNRWVAVPA----VPGA--FVVNVGDL
FHILTNGRFH-----SVYHRA-VV------N--RD-LDRISLGYFLGPPPHAKVAPLR--
-EVVP-------PGR----A-PA---YRAVTW----PEYMGVRKK-AFTTG---------
-------------------------------ASALK--------------------MVAA
AAAATESDDTDAAAAAVHQPPVVVSS------------------------------
>Zm00001d039634_P001

--------------------------------------MPTPSHLNKNPRYLDFRAARRV
PESHAWP---GLHDH------PVVDGGA------------------PGPDAVPVVDLGAA
D--PAP-------------------------APAAAVARAAEQWGAFLLTG--HGV----
PAD--LLARVEDRIATMF-ALPADDKMRAVR-----------GPG-D--ACGYGSP-PIS
SFF--SKCMWSEG------YTF------------------SPASLRADLR----------
--KLWPKAGDDY-TSF------------------------------CDVM-EEFHKHMRA
LADKLLELFLM-AL----G---L--TDEQASAVE------AERRIA-----ETMTATMHL
NWYPRC-PDP---------------------------R-RA--LGLIAHTDSGFFTFVM-
QS---L-VPGLQLF--RH---------A-----PDRWVAVPA----VPGA--FVVNVGDL
FHILTNGRFH-----SVYHRA-VV------N--RD-LDRISLGYFLGPPPHAKVAPLR--
-EAVP-------PGR----A-PA---YRAVTW----PEYMGVRKK-AFTTG---------
-------------------------------ASALK--------------------MVAL
AAAADLDDDGDAAVVHQQQLVVSS--------------------------------
>EES00202

---------------------------------------MPTPSHLANPRYFDFRAARRV
PETHAWP---GLHDH------PVVDGGA------------------PGPDAVPVVDLAGA
A--DEPRA-----------------------AVVAQVARAAEQWGAFLLTG--HGV----

PAE--LLARVEDRIATMF-ALPADDKMRAVR-----------GPG-D--ACGYGSP-PIS
SFF--SKCMWSEG------YTF------------------SPANLRADLR----------
--KLWPKAGDDYTSFC-------------------------------DVM-EEFHKHMRA
LADKLLELFLM-AL----G---L--TDEQVGGVE------AERRIA-----ETMTATMHL
NWYPRC-PDP---------------------------R-RA--LGLIAHTDSGFFTFVL-
QS---L-VPGLQLF--RH---------A-----PDRWVAVPA----VPGA--FVVNVGDL
FHILTNGRFH-----SVYHRA-VV------N--RD-LDRISLGYFLGPPPHAKVAPLR--
-EAVP-------PGR----T-PA---YRAVTW----PEYMGVRKK-AFTTG---------
-------------------------------ASALK-----------------MVALAAA
AAAADLDDDAGAGAAAEPVVHQQLLVSS----------------------------
>KQJ89115

---MPTATHLDFQAARGV
PETHEWP---VLHDH------PVVDGGAGAGED-----------------EVPVVDLRDA
D--PAK-------------------------VAAA-MARAAEQWGAFLLLG--HGV----
PAD--LVARVEERIEAMF-ALPASEKTRAVR-----------GRG-E--SCGYGSP-PIS
GFF--DKSMWSEG------YTF------------------APANVRLDLL----------
--KLWPDAGPHY-------------------------------LLFCEVM-EEFHKGMFA
LAHKLLGLFLS-AL----G---L--TAAD---CA------AAAKIA-----DTMAASMHL
NWYPKC-PDP---------------------------K-RA--VGLIAHTDSGYFTFVL-
QS---M-VPGLQLF--RR--------------EPDRWVAVPA----MPGA--FVINVGDL
FNIVTNGRFH-----NVFHRA-VV------S--RE-SHRVSLGYFLGPPAQAVVAPLD--
-EALT-------LDR----PRPA---YRPVVW----REYMGLREKALF------------
-------------------------------VGESA--------------------LKMI
AVAKDDAPQTPKLHETS---------------------------------------
>KQK02988

---------------------------------------MPTASHLTSPRYFDFRAARGV
PESHAWP---GLHDH------PVVDGGAGAGAG------------EDDADRVPVVDMRAD
P--S---------------------------AAAAAVARAAETWGAFLLEG--HGV----
PES--LLARVEERVAGMF-ALPAPEKMRAVR-----------GRG-E--SCGYGSP-PIS
SFF--AKSMWSEG------YTF------------------SPAHLRRDLR----------
--KLWPKAGPDY-LLF------------------------------CEVM-EEFHREMRL
LADKLLGLFLR-AL----G---L--TADEALAVE------ATHRIA-----ETMTATTHL
NWYPRC-PDP---------------------------K-RA--LGLIPHTDSGYFTFVL-
QS---Q-VPGLQLF--RR---------G---PD--RWVAVPA----VPGA--FVVNVGDL
FSILTNGRFH-----SVFHRA-VV------N--KE-SHRVSLGYFLGPPAQTRVGPLE--
-EALT-------PAR----PKPM---YRPVTW----PEYMTVRKK-AFATG---------
---------------------------------DSA--------------------LQMV
AVDQDDDDDAQTKLANRKIKVSGEAY------------------------------
>LOC_Os05g08540.1

---------------------MQIMTSSSTSPTSPTSPLAAAADNGVAAAYFNFRGAERV
PESHVWK---GMHEK----DTAPVAAADADGGD-----------------AVPVVDMSGG

D--DA--------------------------AVAA-VARAAEEWGGFLLVG--HGV----
TAE--ALARVEAQAARLF-ALPADDKARGAR-----------RPGGG--NTGYGVPPYLL
RYP---KQMWAEG------YTF------------------PPPAIRDEFR----------
--RVWPDAGDDYHRFC-------------------------------SAM-EEYDSSMRA
LGERLLAMFFK-AL----G---L--AGNDAPGGE------TERKIR-----ETLTSTIHL
NMFPRC-PDP---------------------------D-RV--VGLAAHTDSGFFTFIL-
QS---P-VPGLQLLRHRP---------D-------RWVTVPG----TPGA--LIVVVGDL
FHVLTNGRFH-----SVFHRA-VV------N--RE-RDRISMPYFLGPPADMKVTPLV--
-----------AAGS----P-ESKAVYQAVTW----PEYMAVRDK-LFGTN---------
---------------------------------ISA--------------------LSMI
RVAKEEDKES--
>KQL13797

----------------------------------MLSPMTTSPAAAAATPCFELRSAERV
PETHAWP---GIDDH------PTVKAAA-------------------GGDAVPVVDLGGD
P-----------------------------DVARAAAGRAAEEWGAFLLVG--HGV----
AAG--VAARVEEQVARLF-ALPAAEKARAGR-----------RPG-E--FNGYGRAPRLN
-F---SNHMWSEG------YTF------------------PAAAVRAEFR----------
--RVWPDAGDDYLRFC-------------------------------DVM-EEHHAEMRA
LGVRLLDMLFG-AL----G---L--TDAQIAAGE------TEREIR-----ETLTATTHL
NMYPRC-PQP---------------------------E-RA--IGMAAHTDSGFITIIL-
QS---P-VPGLQLL--RR--------------QPDRWVTVPA----PPGA--LVVVLGDL
FQVLTNGRFR-----SALHRA-VV------N--RE-RDRISVPYFLGPPADMKVAPLA--
-AAVP-------PGT----K-AA---FRGVTW----REYLEIREKQAISVD---------
---------------------------------ASV--------------------MKML
QVAEEEEEGGVPPNN---
>Zm00001d037627_P001

---------------------------------MQSSSSSASTPAAASGLVFDLGSAAGV
PETHAWP---GVNEY------PSVESAG--------------------RDVVPVVDMGVA
C--PD--------------------------ATRA-LARAADEWGVFLLVG--HGV----
PRE--VAARAEEQVARLF-VLPAPDKARAGR-----------RPG-EPTATGYGRP-PLA
LRF--SKLMWSEG------YTF------------------RAATVREEFR----------
--RVWPDGGDDYLRFC-------------------------------DVM-EEYDREMRA
LGGRLLDLFFM-AL----GLTDVQFATGE-----------TERRIR-----ETWTATMHP
ILYPRC-PEP---------------------------E-RA--IGLTAHTDSGFITLIM-
QS---P-VPGLQLL--RR---------G-----PDRWVTVPA----PPGA--LIVMLGDL
FQVLTNGRFR-----SPIHRA-VV------S--RE-RERISVPYFLCPPEDMTVAPLA--
-SALL-------PGR----K-AV---FRAVTW----PEYMEVKHK-VFGTD---------
---------------------------------APA--------------------LEML
QLQVDEEEQGERAATT--
>KXG21453

--------------------------------MMPSSSSSASTPAAASGGLFELGSAASV

PETHAWP---GVNEH------PSVESAG--------------------RDAVPVVDMGMG
G--------------PDDADA----------AARA-VARAAEEWGAFLLVG--HGV----
PRG--VAARAEAQVARLF-ALPAPDKARAARRR---------RAA-AAAAAGYGMP-PLA
LRF--SKLMWSEA------YTF-----------------PAAAVRDEFR----------
--RVWPDAGDDYLRFWYVRTPVWYVTRATLPHVDLHLHVHACMRAPSDVM-EEYDREMRA
LGGRLLDLFFM-AL----G---GGLTDDDQIAGGETTT--TERKIR-----DNLTAMMHP
ILYPKC-PEP--------------------------E-RA--MGLAPHTDSGFITLIT-
QS---AGVPGLQLL--RR---------G----PD-RWVTVPA----PPGA--FVVVLGDL
FQVLTNGRYR-----SALHRA-VV------N--RE-RDRISVPYFLGPPDGMKVAPLA--
-SALL-------PGR----RKAA---FRAVTW----PEYMELKHK-VLGTD---------
--------------------------------TSA-------------------LEML
QLDEEEM--
>KQK10911
--MVLQPAQQ
QQPSSQLPYPPHCSERSPAAMDTAPPLVLSPSAPSITDTSTAKAKQSAAPVFDLRREPKI
PAPFVWP---HAEVR------PTTAEEL---------------------GMPVVDVGVL
R--KSRN--------GGGDAA------ALRRAVAQ-VAAAGATHGFFQVSG--HGV----
DAA--LARAALDGASDFF-RMPLADKQRARR-----------VQG-T--VSGYTSA-HAD
RFA--TKLPWKET------LSF-GFHGQD-----------CAEAKPVVVD----------
--YFTSTLGQDF-EPM-----------------------------GRVY-QEYCEKMKE
LSLTIMELLEL-SL----G---V----E----RG------YYREFF-----ADSSSIMRC
NYYPPC-PEP--------------------------E-RT--LGTGPHCDPTALTILL-
QD---D-VGGLEVL--VD---------G-------DWRPVRP----VPGA--MVINIGDT
FMALSNGRYK-----SCLHRA-VV------N--RQ-QERRSLAFFLCPRQDRVVRPPL--
-AVVS-------SSA----P-RQ---YPDFTW----ADLMRFTQR-HYRAD---------
--------------------------------TRT-------------------LDAF
TQWLAPSSSSSSSSSPAPSAAPPPPVASTAQEAA---------------------
>HORVU3Hr1G090980.3
--MVLQTAQQEPSLTR
PPRCSVASAGSPAAMDTSPATPLLLQPPAPSIDPSAAKAAVSKGGGAATAVYDLRREPKI
PAPFVWP---HAEVR------PTTAQEL---------------------AVPVVDVGVL
R--NGD---------AAGIRR----------AVAQ-VASACATHGFFQVSG--HGV----
DNA--LARAALDGASGFF-RLPLAEKQRARR-----------IPG-T--VSGYTSA-HAD
RFA--SKLPWKET------LSF-GFHDRA------------GAAAPVVVD----------
--YFTSTLGPDY-EPM-----------------------------GRVY-QEYCGKMKE
LSLRIMELLEL-SL----G---V----EK---RG------YYRDFF-----ADSSSIMRC
NYYPPC-PEP--------------------------E-RT--LGTGPHCDPTALTILL-
QD---D-VGGLEVL--VD---------G-------DWRPVRP----VPGA--MVINIGDT
FMALSNGRYK-----SCLHRA-VV------N--RR-QERRSLAFFLCPREDRVVRPPP--
-SLRS--------------P-RQ---YPDFTW----ADLMRFTQR-HYRAD---------
--------------------------------TRT-------------------LDAF
TQWFSSAQEAA--
>LOC_Os01g66100.1

------------------MVAEHPTPPQPHQPPPMDSTAGSGIAAPAAAAVCDLRMEPKI
PEPFVWP---NGDAR------PASAAEL----------------------DMPVVDVGVL
R--DGD---------AEGLRR----------AAAQ-VAAACATHGFFQVSE--HGV----
DAA--LARAALDGASDFF-RLPLAEKRRARR-----------VPG-T--VSGYTSA-HAD
RFA--SKLPWKET------LSF-GFHDRA--------------AAPVVAD----------
--YFSSTLGPDF-APM------------------------------GRVY-QKYCEEMKE
LSLTIMELLEL-SL----G---V----E----RG------YYREFF-----ADSSSIMRC
NYYPPC-PEP---------------------------E-RT--LGTGPHCDPTALTILL-
QD---D-VGGLEVL--VD---------G-------EWRPVSP----VPGA--MVINIGDT
FMALSNGRYK-----SCLHRA-VV------N--QR-RERRSLAFFLCPREDRVVRPPP--
-----------SAAT----P-QH---YPDFTW----ADLMRFTQR-HYRAD---------
---------------------------------TRT--------------------LDAF
TRWLAPPAADAAATAQVEAAS-----------------------------------
>KQL08016
--MVSQAQQEPALPHS
SSTAKRAAASLMDARPAQPLLLRAPTPSIDLPASKPDRAAAAAGKAAAASVFDLRREPKI
PAPFVWP---HDDAR------PASAAEL----------------------DVPLVDVGVL
R--NGD---------RAGLRR----------AAAQ-VAAACATHGFFQVCG--HGV----
GAD--LARAALDGASDFF-RLPLAEKQRARR-----------VPG-T--VSGYTSA-HAD
RFA--SKLPWKET------LSF-GFHDGA--------------ASPVVVD----------
--YFAGTLGQDF-EAV------------------------------GRVY-QRYCEEMKA
LSLTIMELLEL-SL----G---V----E----RG------YYRDFF-----EDSRSIMRC
NYYPPC-PEP---------------------------E-RT--LGTGPHCDPTALTILL-
QD---D-VGGLEVL--VD---------G-------DWRPVRP----VPGA--MVINIGDT
FMALSNGRYK-----SCLHRA-VV------N--QR-QERRSLAFFLCPREDRVVRPPA--
------------SGAVGEAP-RR---YPDFTW----ADLMRFTQR-HYRAD---------
---------------------------------TRT--------------------LDAF
TRWLSHGPAQDAPVAAAAST------------------------------------
>Zm00001d012212_P001
MRPRLPPNVPSLPSSLSLLANSLSSPVTNTPTRPDSFPAYLQLAHLMVSQERQEPAVPSS
SSSSAKRAATSMDASPAPPLLLRAPTPSPSIDLPAGKDKADAAASKAGAAVFDLRREPKI
PAPFLWP---QEEAR------PSSAAEL----------------------EVPMVDVGVL
R--NGD---------RAGLRR----------AAAQ-VAAACATHGFFQVCG--HGV----
DAA--LGRAALDGASDFF-RLPLAEKQRARR-----------VPG-T--VSGYTSA-HAD
RFA--AKLPWKET------LSF-GYHDGA--------------ASPVVVD----------
--YFVGTLGQDF-EPM------------------------------GWVY-QRYCEEMKE
LSLTIMELLEL-SL----G---V----EL---RG------YYREFF-----EDSRSIMRC
NYYPPC-PEP---------------------------E-RT--LGTGPHCDPTALTILL-
QD---D-VGGLEVL--VD---------G-------EWRPVRP----VPGA--MVINIGDT
FMALSNGRYK-----SCLHRA-VV------N--QR-RARRSLAFFLCPREDRVVRPPA--
------------SAA----P-RR---YPDFTW----ADLMRFTQR-HYRAD---------
---------------------------------TRT--------------------LDAF
TRWLSHGPAQAAAPPCT---------------------------------------
>Zm00001d042611_P001

--------------MDASPTPPLPLRAPTPSIDLPAGKDRADAAANKAAAVFDLRREPKI
PEPFLWP---HEEAR------PTSAAEL----------------------EVPVVDVGVL
R--NGD---------GAGLRR----------AAAQ-VAAACATHGFFQVCG--HGV----
DAA--LGRAALDGASDFF-RLPLAEKQRARR-----------VPG-T--VSGYTSA-HAD
RFA--SKLPWKET------LSF-GFHDGA--------------AAPVVVD----------
--YFTGTLGQDF-EPV------------------------------GRVY-QRYCEEMKE
LSLTIMELLEL-SL----G---V----E----RG------YYREFF-----EDSRSIMRC
NYYPPC-PVP---------------------------E-RT--LGTGPHCDPTALTILL-
QD---D-VGGLEVL--VD---------G-------EWRPVRP----VPGA--MVINIGDT
FMALSNGRYK-----SCLHRA-VV------N--RR-QERQSLAFFLCPREDRVVRPPA--
------------SAA----P-RQ---YPDFTW----ADLMRFTQR-HYRAD---------
---------------------------------TRT--------------------LDAF
TRWLSHGPAAAAPCT---
>OQU87995
-----------------------MRPRLPPNVPLSLLANSLSSPLPCTLAHLMVSQERQE
PALPLPSNSSSAKRAAASMDASSPAPPLLLRAPTPSPSIDLPAAAGKAAAVFDLRREPKI
PAPFLWP---HEEAR------PTSAAEL----------------------EVPVVDVGVL
R--NGD---------RAGLRR----------AAAQ-VASACATHGFFQVCG--HGV----
DAA--LGRAALDGASDFF-RLPLADKQRARR-----------VPG-T--VSGYTSA-HAD
RFA--SKLPWKET------LSF---------------GFHDGAASPVVVD----------
--YFTGTLGQDFEPMG-------------------------------RVY-QRYCEKMKE
LSLTIMELLEL-SL----G---V----E----RG------YYREFF-----EDSRSIMRC
NYYPPC-PEP---------------------------E-RT--LGTGPHCDPTALTILL-
QD---D-VGGLEVL--VD---------G-------EWRPVRP----VPGA--MVINIGDT
FMALSNGRYK-----SCLHRA-VV------N--QR-QERRSLAFFLCPREDRVVRPPA--
------------SSA----TPRQ---YPDFTW----ADLMRFTQR-HYRAD---------
---------------------------------TRT--------------------LDAF
TRWLSHGPVPAQEAAAPCT-------------------------------------
>LOC_Os05g34854.1
--------------------------------------MHASPHPLQIAHDTLLLSLTHT
LCTAETGSTIRTGTAMVYISNAQDASKLIVTAKGGGGEADDAAASSAAVVLDLWRQPAKI
PAPFVWPRADVALPP----SSPPTGEL----------------------DVPVVDLAAA
L--RD----------AAGMRR----------AVAQ-VAAACASHGFFQVSG--HGV----
PPS--LARAALDGAAGFF-RLPPAAKQRARR-----------APG-T--VTGYTAA-HAD
RFV--DNLPWKET------LSF-GHRHAN-----------AAGNNSSTVA----------
--DYFSTLGDDF-KHL------------------------------GEVY-QEYCEAMEE
VTKAIMAVLGE-SL----G---V----G----GG------YYREFF-----EDSSSIMRC
NYYPPC-PEP--------------------------E-RT--LGTGPHCDPSALTVLL-
QD---GDVDGLQVL--VA---------G-------AWRPVRP----LPGA--FVVNIGDT
FMALTNGRYK-----SCLHRA-VV------H--RE-QERRSLAFFLCPREDRVVRPPA--
--GAG-------AGE----R-RL---YPDFTW----ADFMRFTQR-HYRAD---------
---------------------------------TRT--------------------LDAF
ARWLRPPACSGAAPVVGPPTTATQAATV----------------------------

>EES19547

-------MPENPHHGSSDRLALVGKQVDRRLLLPVSPAADDGKDDNGAAAVINLWGQLKI
PDPFVWS---HAETM------VSSEREL----------------------DAPVVDVGAA
M--RGGDDDC-----GAGIRR----------AAEL-VSGACSSHGLFQVTG--HGL----
DPA--LARAALDGAAAFF-RLPLATKQRASR-----------APG-N--VTGYTAA-HAD
RFT--ANLPWKET------LSF-------------GHRDRRTSGSHVVVD----------
--YFTSTLGSDF-KPL-------------------------------GVVYQNYCNAMKE
VSLAIMEVIGV-SL----G---V----G----RS------YYRDFF-----ADGSSIMRC
NYYPPC-PEP---------------------------E-RT--LGTGPHCDPSALTVLL-
QD---GDVDGLQVL--VD---------G-------EWRTVRP----RPGA--LVVSIGDT
FMALSNGRYR-----SCLHRA-VV------H--RE-RERRSLVFFLCPREDRVVRPPP--
RLLLAVAAREQQQEQ----PRRR---YPDFTW----ADLARFTQR-HYRAD---------
---------------------------------AGT--------------------LDAF
ARWLGAAPTCAAATSAASQSPDKAHETV----------------------------
>KQK06181

---------MVDYQLSNSGEPNATTPIAAMDQVGRNPLLLFGPADDSKNAIIPWRQSKQI
PDSFVWP---HADTH------PPSSSTTTTTEL----------------LDVPVVDLAAA
L--RD----------AAGMRD----------AAAQ-AAAACASHGFFLVTG--HGV----
DPA--LARAALDGAAGFF-RLPLATKQRARR-----------VTG-S--VAGYAAA-HAD
RFA--ANLPWKET------LSFRHHHDDD--------------DRDAVLD----------
--YFTSTLGDDF-KPL------------------------------GEVY-QEYCGAMEA
ASLAIMEVLGV-SL----G---V----G----RG------HYRDFF-----ADGSSVMRC
NYYPPC-PEP---------------------------D-RT--LGTGPHCDPSALTLLM-
QD---GGVDGLQVL--VD---------G-------GWRPVRP----KPDE--LVVNIGDT
FMALSNGRYK-----SCLHRA-VV------H--RE-RERRSLAYFLCPRKDRVVRPPP--
-STT--------APA----P-RL---YPDFTW----ADLSRFTQR-HYRAD---------
---------------------------------ART--------------------LDAF
ARWLGPAKAQEIV---
>HORVU1Hr1G063780.2

---MVDVSNFVEANGNAAVSIPAMEVAGSPHVPFVPRDANATDSKNAKDVLDLWRQQKQI
PAPFIWP---HADAR------PSSILEL----------------------DVPVVDIGAA
L--HS----------AAGMAR----------AAAQ-VAEACASHGFFQVTG--HGV----
DPA--LAQAALDGAADFF-RLPLATKQRARR-----------SPG-T--VKGYASA-HAD
RFA--AKLPWKET------LSFIHNHVHE---------DVGARASSHVVD----------
--YFTSALGDDFMHLG-------------------------------EVY-QEYCEAMED
ASLAIMEVLGV-SL----G---L----G----RG------YYRDFF-----ADGSSIMRC
NYYPRC-PEP---------------------------D-RT--LGTGPHCDPSALTILL-
QD---GEVDGLQVL--VD---------G-------AWRSVRP----KPGE--LVVNIGDT
FMALSNGRYK-----SCLHRA-VV------H--RE-KERRSLAYFLAPQEDRVVRPPP--
----------SPAPA----P-RL---YPDFTW----AELMRFTQR-HYRAD---------
---------------------------------ART--------------------LDAF

ACWLDLPSCPATPQAQGTV---
>HORVU3Hr1G089980.1

---MASLVFDAAVLSRKEDI
PPQFIWP---ADEAP------SVDGVEE---------------------IVVPVVDLAGF
L--AGDD------------------------AGLNELVAACERHGFFQVVN--HGV----
DPA--LLAKAYRCCDAFY-ALPLAEKQRAQR-----------RLG-E--NHGYAGS-FVG
RFG--SKLPWKET------MSF--------------NCSAAPESARKVVD----------
--YFVGVLGEEY-RHM------------------------------GDVW-QEYCNEMTR
LALDVTEVLAA-CL----G---L----D----RG------ALRGFF-----AGDDSLMRL
NHYPPC-KKP---------------------------H-LT--LGTGPHHDPTALTLLH-
QD---D-VGGLEVF--TG---------G-------AWRAVRP----RSDA--FVVNIGDT
FSALTNGRHV-----SCLHRA-VV------N--GS-LARRSLTFFLNPQLDRPVTPPA--
-ELLAI------DGR----P-RV---YPDFTW----REFLEFTQK-HYRSD---------
---------------------------------SRT--------------------LDAF
VAWINQGHTG--
>KQL22579

--------------------------MAAPAGADEPCSAAQACSMAVVFDAEVLSREESI
PAQFVWP---PEERA------PAAGLGV-------------------EEMDIPVVDLAEF
---------------LRGGGE----------LPVG-MAEACERHGFFQVVG--HGV----
GAE--LIAEAYRCCDAFY-AHPLAEKQRARR-----------LPG-E--SHGYASS-FTG
RFD--SRLPWKET------LSF--HCPAA-------AAAPGSAGGRAVAD----------
--YIVGVLGEEY-RHM------------------------------GEVY-QEYCDAMTR
LALDVTEVLAA-AL----G---L----PD---RG------VLRGFF-----AGGDSVMRL
NHYPPC-RQP---------------------------H-LT--LGTGPHRDPTSLTLLH-
QD---C-VGGLQVR---A---------GGDGGGGGEWLAVRP----RADA--FVVNIGDT
FAALTDGRHA-----SCLHRA-VV------S--GG-AARRSLAFFLNPPLDRVVCPPE--
-ALLP-------AGR----P-RA---YPDFKW----RELLEFTQK-RYRSD---------
---------------------------------AST--------------------MDAF
VSWIARGRGDGGGGGHDHGGQEEEK-----------------------------------
>Zm00001d007894_P001

---MAAAAVVFDAEALSREEHI
PAQFVWP---TEERA------PAGGVEE---------------------VAIPVVDLGEF
L--RRGV------------------------LPRG-VAEACERHGVFQVVN--HGV----
GAA--LLAEAYRCCDAFY-ALPLADKQRAQR-----------RHG-E--NHGYASS-FTG
RFH--CCLPWKET------LSF--------------NCPAGAGTARAVVG----------
--YFVDVLGEDY-RHMGSLLLYPIFLRACACHAARTETGKKCR----EVY-QEYCDAMTR
LALDVTEVLAA-AL----G---L----D----RG------ALRGFF-----EGGDSVMRL
NHYPAC-RQP---------------------------H-LT--LGTGPHRDPTSLTLLH-
QD---D-VGGLQVR--AG---------G------GPWRAVRP----RADA--FVVNIGDT
FAALTDGRHT-----SCLHRA-VV------T--GG-GSRRSLAFFLNPPLDRVVRPPG--
-ALLQENKQ---AGR----P-RA---FPDFTW----REFLEFTQK-HYRSD---------

---------------------------------AGT--------------------MDAF
VSWIAGGRRHHGGQEEGN--------------------------------------
>EER95912

--MAAAGVVVFDAEVLSREERI
PAQFVWP---AEDRA------PSAGGCG------------------VEEIAIPVVDLGEF
L--------------RHGDRE----------LPRG-VAEACERHGFFQVVN--HGV----
PAA--LLADAYRCLDAFY-ARPLADKQRAQR-----------RPG-E--SHGYASS-FTG
RFR--SCLPWKET------LSF---NCPA-------GTGGDTQSAAAVVD----------
--YFVDVLGEDY-RHMG------------------------------EVY-QEYSDEMAR
LAMDVTEVLAA-AL----G---L----R----RG------ALRGFF-----DGGDSIMRL
NHYPAC-RQP---------------------------Q-LT--LGTGPHRDPTSLTLLH-
QD---D-VGGLQVC--AG---------G---GGGGEWRAVRP----RADA--FVVNIGDT
FAALTDGRHA-----SCLHRA-VV------S--GD-RARRSLAFFFNPPLDRVVRPPD--
-ALLL-------EDEKRRRP-RA---FPDFTW----REFLEFTQK-HYRSD---------
---------------------------------AST--------------------MDAF
VSWIAGGRGDGHGGRQEGK-------------------------------------
>LOC_Os07g07420.1

---MAAVVFDAAILSKQEAI
PAQFVWP---ADEAP------AADDGVV-------------------EEIAIPVVDLAAF
L--ASGG------------------------IGRD-VAEACERHGFFQVVN--HGV----
DPA--LLAEAYRCCDAFY-ARPLAEKQRARR-----------RPG-E--NHGYASS-FTG
RFD--CKLPWKET------MSF--------------NCSAAPGNARMVAD----------
--YFVDALGEEY-RHM------------------------------GEVY-QEYCDVMTR
LALDVTEVLAV-AL----G---L----G----RG------ELRGFF-----ADGDPVMRL
NHYPPC-RQP---------------------------H-LT--LGTGPHRDPTSLTLLH-
QD---D-VGGLQVL--PD---DAAAAAG-------GWRAVRP----RADA--FVVNIGDT
FAALTNGRHA-----SCLHRA-VV------N--GR-VARRSLTFFLNPRLDRVVSPPP--
-ALVD-------AAH----P-RA---FPDFTW----REFLEFTQR-HYRSD---------
---------------------------------TNT--------------------MDAF
VAWIKQRNGYESLDKY--
>KQK20711

----VWP---ADEAP------PTTVEEI----------------------SIPVIDLAAF
L--S-----------GSGDFS----------GEDH-FAAACERHGFFQIVN--HGV----
DPS--LLAEAYRCMDAFY-ALPLAEKQRAQR-----------RLG-E--NHGYAGS-FTG
RFE--SRLPWKET------MSF-NYSDAP-------G------NASKVVD----------
--YFVSVLGEEY-RQM------------------------------GEVW-QGYCDVMTR
LAQDVTDLLAV-GL----G---L----G----RG------GLRAFF-----AGGDSVMRL
NNYPPC-RQP---------------------------H-LT--LGTGPHRDPTSLTLLH-
QD---L-VGGLQVF--VG---------G-------EWRAVRP----REDA--FVVNIGDT
FAALVDGRHA-----SCLHRA-VV------N--GA-AARRSLTFFLNPPLDRVVSPPP--

-GLLLAEDVDG-GAI----N-RK---YPDFTW----REFLEFTQK-HYRSD---------
--------------------------------TNT---------------------MDAF
VDWIKQGRR--
>KQK20710

--MALEFDATILSRAPVI
PPQFVWP---ADEAS------PAAVGEI----------------------AIPVIDLAAF
L--S-----------GSGDFS----------GADH-FAAACERHGFFQIVN--HGV----
DPA--LLAEAYRSMDAFY-ALPLAEKQRAKR-----------RLG-E--NHGYAGS-FTG
RFE--SRLPWKET------MSF-NCSDAP-------E------NASKVVD----------
--YFVSVLGEEY-RQM------------------------------GEVW-QEYCDVMTR
LALDVTDLLAV-GL----G---L----G----RG------ALRGFF-----AGGDSVMRL
NNYPPC-RQP---------------------------H-LT--LGTGPHRDPTSLTLLH-
QD---L-VGGLQVF--VG---------G-------EWRAVRP----REDA--FVVNIGDT
FAALVDGRHA-----SCLHRA-VV------N--GA-AARRSLTFFLNPPLDRVVSPPQ--
-GLLVAKDG---GAI----N-RK---YPGFTW----REFLEFTQK-HYRSD---------
--------------------------------TNT---------------------MEAF
VDWIKQGRRGAQFHGDAGQAEKN--------------------------------
>LOC_Os03g63970.1

------------------------------------MSMVVQQEQEVVFDAAVLSGQTEI
PSQFIWP---AEESP------GSVAVEE---------------------LEVALIDVGAG
AERSS--------------------------VVRQ-VGEACERHGFFLVVN--HGI----
EAA--LLEEAHRCMDAFF-TLPLGEKQRAQR-----------RAG-E--SCGYASS-FTG
RFA--SKLPWKET------LSF------------RYSSAGDEEGEEGVGE----------
--YLVRKLGAEHGRRL------------------------------GEVY-SRYCHEMSR
LSLELMEVLGE-SL----G---I--VGDR---RH------YFRRFF-----QRNDSIMRL
NYYPAC-QRP---------------------------L-DT--LGTGPHCDPTSLTILH-
QD---H-VGGLEVW--AE---------G-------RWRAIRP----RPGA--LVVNVGDT
FMALSNARYR-----SCLHRA-VV------N--ST-APRRSLAFFLCPEMDTVVRPPE--
-ELVD-------DHH----P-RV---YPDFTW----RALLDFTQR-HYRAD---------
--------------------------------MRT---------------------LQAF
SDWLNHHRHLQPTIYS---------------------------------------
>KQK86097

----------------------------MVQAALDDTKQQQQQPPLVFDAARLSGQADI
PEQFLWP---ADESP------TPDAVEE---------------------LPVPLIDLSG-
---------------GAANAE----------VVRQ-VGDACGLHGFFQVVN--HGI----
DPA--LLAEAHRCMDTFF-TLPLADKQRAQR-----------RPG-E--SCGYASS-FTG
RFA--SKLPWKET------LSF-RYSPSD-----------DVAGEQLVSS----------
--YFVEKLGEAY-RHH------------------------------GEVY-GRYCSEMSR
LSLEIMEVLGE-SL----G---V----G----PS------HFRRFF-----EGNDSIMRL
NYYPPC-QRP---------------------------Y-DT--LGTGPHCDPTSLTILH-
QD---D-VGGLQVF--AG---------G-------RWRSIRP----HAGA--FVVNIGDT

FMALSNGRYR-----SCLHRA-VV------N--SR-VPRRSLAFFLCPEMDKVVRPPG--
-ELVD-------DRN----P-RA---YPDFTW----RALLDFTMR-HYRAD---------
---------------------------------MRT--------------------LEAF
SNWLLIHGNNSHQL--
>Zm00001d034898_P001

---MAAHWQAGRAGTSKQAVAVAAASSSASSLLLLTMVLAAHDPPPLVFDAARLSGLSDI
PQQFIWP---ADESP------TPDSAEE---------------------LAVPLIDLSGD
A--AE--------------------------VVRQ-VRRACDLHGFFQVVG--HGI----
DAA--LTAEAHRCMDAFF-TLPLPDKQRAQR-----------RQG-D--SCGYASS-FTG
RFA--SKLPWKET------LSF-RYTDDD----------DGDKSKDVVAS----------
--YFVDKLGEGY-RHH------------------------------GEVY-GRYCSEMSR
LSLELMEVLGE-SL----G---V----G----RR------HFRRFF-----QGNDSIMRL
NYYPPC-QRP---------------------------Y-DT--LGTGPHCDPTSLTILH-
QD---D-VGGLQVF-------------D---AATLAWRSIRP----RPGA--FVVNIGDT
FMALSNGRYR-----SCLHRA-VV------N--SR-VARRSLAFFLCPEMDKVVRPPK--
-ELVD-------DAN----P-RA---YPDFTW----RTLLDFTMR-HYRSD---------
---------------------------------MRT--------------------LEAF
SNWLSTSSNGGQHLLEKK--------------------------------------
>EER90481

---------------------------MVQQASLGEPLLLQPPPPSLVFDAARLSGLSDI
PQQFLWP---ADESP------TPDAAEE---------------------LAVPLIDLSGD
A--AE--------------------------VVRQ-VRRACDLHGFFQVVN--HGI----
DDA--LLQEAHRCMDAFF-TLPMSDKQRAQR-----------RQG-D--SCGYASS-FTG
RFA--SKLPWKET------LSF--------------RYSDDQGDGDVVVD----------
--YFVDKLGDAYRHHG-------------------------------EVY-GRYCSEMSR
LSLELMEVLGE-SL----G---V----G----RR------HFRRFF-----QGNDSIMRL
NYYPPC-QRP---------------------------Y-DT--LGTGPHCDPTSLTILH-
QD---D-VGGLQVFDAAT---------G---PGTGRWRSIRP----HPGA--FVVNIGDT
FMALSNGRYR-----SCLHRA-VV------N--SR-VPRRSLAFFLCPEMDKVVRPPA--
-ELVD-------DAN----P-RA---YPDFTW----RTLLDFTMR-HYRSD---------
---------------------------------MRT--------------------LEAF
SNWLNHGGHLSSPPPP--
>KQK12006

---MVMQPVLFDAAVLSGRSDI
PSQFIWP---ADESP------TPDAAEP---------------------LDVPLIDIGGI
V--AS----------GAGDRA------AAVASVARLVGDACSRHGFFQVVN--HGI----
DAA--LLADAHRCVDAFF-KLPLAEKQRALR-----------RPG-E--SCGYASS-FVG
RFS--SKLPWKET------LSF----------------RSSPSCPDLPLD----------
--FILSNLGEEH-RRL------------------------------GEVY-ARYCEEMSR
VSLEIMEVLGE-SL----G---V----G----RS------HYRSFF-----EGNDSIMRL
NYYPPC-QRP---------------------------Y-ET--LGTGPHCDPTSLTILH-

QD---A-VGGLQVH--VD---------G-------RWRAIAP----RQDA--FVVNIGDT
FMALSNGRYK-----SCLHRA-VV------N--SK-VPRKSLAFFLCPEMDKTVAPPG--
-KLVD-------EEN----P-RV---YPDFTW----RALLDFTQK-HYRAD---------
--------------------------------MKT--------------------LEVF
SGWVLQQQKQKQLGS---
>HORVU5Hr1G124120.2

--MVQPVFDAAVLSGRTDI
PSQFIWP---EGESP------TPDATEE---------------------MHVPLIDIGGM
L--SGD---------PRAAAE----------VTRL-VGEACERHGFFQVVN--HGI----
DAQ--LLADAHRCVDAFF-TMPLPEKQRALR-----------RPG-E--SCGYASS-FTG
RFA--SKLPWKET------LSF----------------RSCPSDPALVVD----------
--YIVATLGEDHRRLG-------------------------------EVY-ARYCSEMSR
LSLEIMEVLGE-SL----G---V----G----RA------HYRRFF-----EGNESIMRL
NYYPPC-QRP--------------------------L-ET--LGTGPHCDPTSLTILH-
QD---D-VGGLQVH--TD---------G-------RWRSIRP----RADA--FVVNIGDT
FMALSNGRYK-----SCLHRA-VV------N--SR-VPRKSLAFFLCPEMDKVVAPPG--
-TLVD-------AAN----P-RA---YPDFTW----RSLLDFTQK-HYRAD---------
--------------------------------MKT--------------------LEVF
SSWVVQQQPASART---
>LOC_Os01g22910.1

---------MGWDEPTFRGVWLRGEWDGLVPGEEYSSQIRDQLIGENVADELVPPGTSER
RDDAFLP---RSRCS------PSGATAD---------------------GSGDGDSGRL
G--DGDHAGLGDDDRGGGASAMARRIQWWGGQRRL-IRRWGGLWRWIRRRG---GA----
AAA--CEEEVEGRRRIRRWGLRWLARRSSRGRS---------GGGVRGGGRGAAADPAMG
GAA--TAFPLALAHRRLFLLLLLAAGSSSCSSSGDGEDCGGRSGDRLTES----------
--LFVDIGLESDCR---------------------------------QVV-NDYVEAVRQ
LACHVLDLLGE-GL----G---L----RD---PT------SLTRLI---TATDNDSLIRI
NHYPPSCAAAAGDHKS---------------GGGPAPT-AA--IGFGEHTDPQILSVLR-
AN---D-ADGLQLL--LP---DAAAA-G----DS-VWVPVPP----DPSA--FFVNVGDL
LQALTNGRLV-----SIRHRV-VV------G--TG-KPRLSTIYFAAPPLHARISALP--
-ETVA-------AGA----P-RR---YRAFTW-------AEYKRT-MYTLR---------
-------------------------------LSHNR--------------------LDLF
HAGDGDGDAGVGDDDDHE--------------------------------------
>LOC_Os05g06670.1

------------------------------------MVVPSATTPARQETVVAAAPPAA
AASGVVG---GGGGV-----------------------------------TIATVDMSAE
R--GA--------------------------VARQ-VATACAAHGFFRCVG--HGV----
PAAAPVAARLDAATAAFF-AMAPAEKQRA-------------GPA-S--PLGYGCR-SIG
-FN--GDVGELEY------LLL----------------HANPAAVAHRAR----------
--TIDAMDPSRF-----------------------------------SAIVNEYIEAMKK
LACEILDLLGE-GL----G---L----KD---PR------YFSKLT---TNADSDCLLRI

NHYPPS-CNIHKLDHDDQCNIKSLVSTKASNGGNLMAG-GR--IGFGEHSDPQILSLLR-
AN---D-VEGLQVF--VP------DHEG----KE-MWVQVPS----DPSA--IFVNVGDV
LQALTNGRLI-----SIRHRV-IA------T--AC-RPRLSTIYFASPPLHARISALP--
-ETIT-------ASS----P-RR---YRSFTW-------AEYKTT-MYSLR---------
-------------------------------LSHSR--------------------LELF
KIDDDDSDNASEGKA---
>KQK99291

---TVV-NEYVGAVRQ
LACEILDLLGE-GL----G---L----KD---PR------SFSKLI---TDTDSDSLLRI
NHYPPA-CTIHKLDHDGQCKMKSIVRTKNGNGVNPSAG-AR--IGFGEHSDPQIISLLR-
AN---D-VNGLQVL--LP------NSDG----KE-VWIKVPA----DPSA--FFVNVGDL
LQALTNGKLV-----SVRHRV-IA------S--AC-RPRLSTIYFAAPPLHAQISALP--
-ETIT-------AAS----P-RQ---YRSFTW-------AEYKKT-MYSLR---------
-------------------------------LSHSR--------------------LDLF
HVGDDDNSNVGKGEQE--
>Zm00001d024175_P001

--MVVPSTTPVV
RQQTTPP--QSSHAG-----------------------------------GIPTVDLSAH
GGRGA--------------------------LSRQ-VVRACAEHGFFRAVN--HGV----
PPG--PAARLDAAARTFF-ALAPRDKQRA-------------GPP-S--PLGYGCR-SIG
-FN--GDAGELEY------LLL---------------HANNPAAVAHRAR----------
--AIDAEEPSRF----------------------------------SNVV-NEYVGAMRQ
LACEILDLLGE-GL----G---L----ED---PR------SFSKLI---TDTDSDSLLRI
NHYPTA-CNAHNLDHDSQCKMKSSVRTKTTSNGVKPSAGGR--VGFGEHSDPQILSLLR-
AN---D-VDGLQVLLNAD---------G-----KEVWVQVPA----DQSA--FFVNVGDL
LQALTNGKLV-----SVRHRV-IA------S--SS-RARLSTIYFAAPPLHARILALA--
-ETIT-------ANA----P-SQ---YRPFTW-------AEYKKT-MYSLR---------
-------------------------------LSHSR--------------------LNLF
HIDHDGHSNVGEGEE---
>Zm00001d037565_P002

--MVVPSTTPVV
RQETPPP---SHDGI-----------------------------------GIPTVDLSAP
GGRGA--------------------------LSRQ-VARACAQHGFFRAVN--HGV----
APG--PAARLDAAARTFF-ALAPHNKQRA-------------GPP-S--PLGYGCR-SIG
-FN--GDAGELEY------LLL----------------HANPAAVAHRAR----------
--SIDTDDPSRF----------------------------------SNVV-NEYVGAMRQ

LACEILDLLGE-GL----G---L----KD---PR------SFSRLI---ADTDSDSLLRI
NHYPPP-CAIHKLDHDSQCRMKNSFRIVAGNGANQSAG-AR--IGFGEHSDPQILSLLR-
SN---D-VDGLQVLLNSD---------G-----REVWVQVPA----DPSA--FFVNVGDL
LQAFTNGKVI-----SVRHRV-IA------S--SS-RARLSTIYFAAPPLHARILALP--
-ETVT-------ANS----P-RQ---YRPFTW-------AEYKKT-MYSLR---------
------------------------------LSHSR--------------------LNLF
HIGHDDHGKGEQE---
>OQU77483

---MVVPSTTPVVV
RQETPPP---PLPLP------PSHDGII----------------------GIPTVDMSAP
GGRGA--------------------------LSRQ-VARACAEHGFFRAVN--HGVAVAP
AAG--PAARLDAAARTFF-ALPPHDKQRA-------------GPP-S--PLGYGCR-TIG
-FN--GDAGELEY------LLL----------------HANPAAVAHRAR----------
--SIDTDDPSRF----------------------------------SNVV-NEYVGAMRQ
LACEILDLLGE-GL----G---L----KD---PR------SFSKLI---TDTDSDSLLRI
NHYPPA-CTIQKLDHDNQCKMKSSFRIKTGNGVNQSAG-AR--IGFGEHSDPQILSLLR-
SN---D-VDGLQVLLNCD---------G-----REVWVQVPS----DPSA--FFVNVGDL
LQALTNGKVI-----SVRHRV-IA------N--SC-RARLSTIYFAAPPLHARILALP--
-ETIT-------ANS----P-RQ---YRPFTW-------AEYKKT-MYSLR---------
------------------------------LSHSR--------------------LNLF
HIDHDDHSNVGKGEQE--
>KQK07366

--MVVPSSTTPARQ
EIATAAA---ALQLP-----------------------------------AIPTVDMSAP
R--GL--------------------------LSRQ-VARACAEQGFFRAVN--HGVLVPG
PAP--AARQLDAATAAFF-ALPAHEKQRARGVQGQ-------GPPSS--PLGYGCR-TIG
-FS--GDVGELEY------LLL------------------HANDPAYKAA----------
--SIHAHDPKHF-----------------------------------SCVVNEYVEAVKQ
LACDILDLLGE-GL----G---L----ED---PR------SFSKLI---TEPDSDSLLRI
NHYPPS-CTVHKLDHDGQCKLKGTACRAKAGNGGNPTGGGR--IGFGEHSDPQILSLLR-
AN---D-VDGLQVL--LP------DVNG-----KEVWIQVPA----DSSA--FFVNVGDL
LQALTNGKLL-----SVRHRV-IA------S--AC-RPRLSTIYFAAPPLHARISALP--
-ETVT-------AGS----P-RR---YRSFTW-------AEYKTT-MYSLR---------
------------------------------LSHSR--------------------LDLF
CVHDDDDNSSDVSKEK--
>HORVU1Hr1G023460.3

---RQE
TATLLLP---QAQPS------PGGG------------------------AIPTVDMSAP
RGRGA--------------------------LSRQ-VARACAEQGFFRAVN--HGV---P
PAG--PPARLDAATSAFF-ALAAHDKQRA-------------GPP-S--PLGYGCR-SIG
-FN--GDVGELEY------LLL----------------HANPAAVAHRAS----------

--SIDTNDPSRF----------------------------------SSVV-NEYVEAVKQ
LACDILDLLGE-GL----G---L----ED---PR------LFSKLV---TEADSDSLLRI
NHYPTKAGNGANPGAG-----------------------GR--IGFGEHSDPQLLSLLR-
AN---D-VDGLQVL-LPD-------VNG-----KDAWIQVPA----DSSA--YFVNVGDL
LQALTNGRLV-----SIRHRV-IA------S--AC-RPRLSTIYFAAPPLHARISALP--
-EMVT-------ASS----P-RR---YRSFTW-------AEYKTA-MYSLR---------
-------------------------------LSHSR--------------------LDLF
HVDDDESGNA--
>KQL05037

--MVVPSAASRDMA
PESLPLG---IIPTVDMSAP
SGRGD--------------------------LARR-LVRACAERGFFKAVN--HGV----
PAR--AAARLDAAASAFF-ARPARAKQAA-------------GPP-D--PLGYGSR-NIG
-GN--GDVGELEY------LIL----------------HADPAAVARKAT----------
--VIDAEDPSRF-----------------------------------SVAVNEYVDAVRR
LACRVLDLLGE-GL----G---F----RD---PT------SLSQLI---SAVDSDSLLRI
NHYATSAAAL------------------QGPGGRSCTK-GS--IGFGEHTDPQILSVLR-
AN---D-VDGLQVL-LPD---------G--CGGGDEWVPVPA----DPAA--FFINVGDL
LQALTNGRLV-----SIRHRV-MA------S--TT-RPRLSTIYFAAPPLRALIAALP--
-ETVA-------AGT----P-RR---YRPFTW-------AEYKKA-MYAHQ---------
-------------------------------LSHNR--------------------LDLF
HASSDDAADDGHS---
>Zm00001d040737_P001

---MVAPSAAAGRDMA
PESLPLG---IIPTVDMSAP
SGRGE--------------------------LARR-LVRACAERGFFKAVN--HGV----
PPR--VSARLDAATSAFF-ARPAPVKQAA-------------GPP-D--PLGYGSR-NIG
-AN--GDVGELEY------LIL----------------HAQPAAVARKAM----------
--VIDAEDPSRF-----------------------------------SVAVNEYVDAVRR
LACRVLDLLGE-GL----G---L----GD---PT------SLSRFV--SAVVDSDSVLRI
NHYPTS-SAAADVS-----------------------A-KG--IGFGEHTDPQILSLLR-
AN---D-VDGLQVL--LP---DARGGSG----GD-QWVQVPA----DPSA--FFINVGDA
LQALTNGRLV-----SIRHRV-MA------S--TT-RPRLSTIYFAAPPLDARIAALP--
-ETVV-------AGA----A-RR---YRTFTW-------AEYKKA-MYALR---------
-------------------------------LSHNR--------------------LDLF
HADASSGGSGDKKHDI--
>EES02896

--MVVPSAAGREMA
PESLPLG---IIPTVDMSAP
C--GE--------------------------LARR-LVRACAERGFFKAVN--HGV----
PPR--VSARMDAAASAFF-ARPGQAKQAA-------------GPP-D--PLGYGSR-NIG

-AN--GDVGELEY------LIL----------------HADPGAVARKAK----------
--VIDKDDPSRF-----------------------------------SVAVNEYVGAVRH
LACRVLDLLGE-GL----G---L----RD---PT------SLSRLI---SAVDSDSLLRI
NHYPTSRSSAADI------------------------STKG--IGFGEHTDPQILSLLR-
AN---D-VDGLQVL-LPD---------G-HGGGDEQWVQVPA----DPSA--FFINVGDL
LQALTNGRLV-----SIRHRV-MA------S--TT-RPRLSTIYFAAPPLDARVAALP--
-ETVT-------AGA----P-RR---YRTFTW-------AEYKKA-MYALR---------
-------------------------------LSHNR--------------------LDLF
HATSGSSRDVIDDDHEQ---------------------------------------
>KQK04223

---MVMPASTASSAAC
RDTASGA---GIPTVDMSAP
WGRAE--------------------------LSRQ-MVEAFAERGFFKAVN--HGV----
PPR--APATLDAATAAFF-ERPAPEKQAA-------------GPP-D--PLGYGSR-SIG
-SH--GDVGELEY------LTL----------------HADPAAVARRAV----------
--AIDREDPSRF----------------------------------SEAV-NEYVGAVRR
LACQILDLLGE-GL----G---L----ED---PT------ALSKII---TVSDSDSIIRI
NHYPPSSAAA-----------------------------SS--VGFGEHSDPQILSVLR-
AN---D-VDGLQVL-LPD---------G---RGQDTWVQVPA----DPAA--FFINVGDL
LQALTNGRLV-----SIRHRV-MA------S--TS-KPRLSTIYFAAPALHALISALP--
-ETVT-------SDA----P-RR---YRPFTW-------AEYKKN-MYTLS---------
-------------------------------LSHNR--------------------LELY
NAVNADAGEARPRSGIAS--------------------------------------
>HORVU7Hr1G101720.1

---ASSSAAAVA
PACR------GMAPA-----------------------------------GIPTIDMSAP
AGRAE--------------------------MSRQ-MVEAFAERGFFKAVN--HGV----
PPR--ASARLDAASAAFF-ARPAEEKQEA-------------GPP-D--PLGYGSR-SIG
-SH--GDVGELEY------LIL----------------HTDPEVVARKAR----------
--AVDRDDPSRF----------------------------------SEAV-NEYVQAVRH
LACRILDMLGE-GL----G---L----RD---PA------SLSRLI---TTTDGDSLVRI
NHYPASAAGDGGVK-----------------------AASS--VGFGEHTDPQILSVLR-
AN---D-VDGLQVL--LP---DGR---G----ED-AWVQVPA----DPAA--FFINVGDL
LQALTNGKLV-----SIRHRV-MA------S--SS-KPRLSAIYFAAPALHERISAFP--
-ETVT-------AAA----P-RR---YRPFTW-------AEYKKT-MYTLR---------
-------------------------------LSHNR--------------------LDLF
AVVGDSGEDKPRI---
>HORVU3Hr1G029010.7

-----------------------------------GGPCQGRAGADSPAGGAAAAGPRAG
DRPVRRP---GARPR------RRGARAG------------------VRVRGAGLLQGDRP
R--G---------------------------AAGARARRGGGRGGVLRAAAGREGG----

RRR--PAARVRQQADRRL-RRPRVDR----------------VPPAGRHPRRRAAR-RLL
RVLDIAVRGRRRG------VVV---------------RAPLPLTVSSSVR----------
--SDLL-EEYAAAVRR
MACGVLELMAE-GL----G---I----GP---AD------ALSRLV---SDGESDNMLRV
NHYPPR-PEMQG-------------------------R-LL--TGFGEHTDPQIISVLR-
SN---G-TSGLEIC-ARD---------G-------EWTSVPP----DPDA--FFVNVADA
LQVLTNGRFS-----SVKHRG---------------------------------------
------------------------------WW----------------------------

>LOC_Os05g11810.1

---MAQPEKEAAAA
A------------------------------AAAA-------------------------
--VPG-S--PFGYGSK-RIG
-CN--GDLGWVEY------LLL-----------------GVAAAAAAPLP----------
-AHGEASPSPSYGSFR-------------------------------DIL-NEYVVAVRA
MMWEVLKLMAE-GL----G---L----KE---KD------ALVRLV---SHEESDSVLRV
NHYPPH-PELKQQGHG-----------------------RL--TGFGEHTDPQIISVLR-
SN---D-TSGLEIS-LRD---------G-------SWASVPP----DRKS--FFVNVGDV
LQ--PGGRIKLEELQGFLHAV-SLRSRATREATRS-RPAAGLLSLASPTSPPPEQPPA--
-KPDGGK-----DGG----G-GA---FYPLQHPAVGAPTGNRRRG-GDASRRLRRRRRPE
AAEPASLLADLAPSQPDRDEVAPAALHQDEALAGAV--------------------VAPA
EKAAGWGSRGAGGGDVAGGGDEAASLGAAGSGSPRPDPTPAAGPRRRPAPGRRRRR
>KQK03260

--MVVLAKGEL-
-EQIALP---AAQSP---------PA------------------------NVQAVDLSSA
RRSGPGRAA----------------------AARA-LVAACEEQGFFKVTG--HGV----
APA--LVRALDAAAAAFF-ALPQAEKEAA-------------AGR----PVGYASK-RIG
-TA--GDLGWIEY------LLL--CVAPGAGQALPVPASASFSTLPCAAA---AAAAAAS
LSGSEPESSCPL-R---------------------------------GLL-EEYAAALRR
MACEVLELMAE-GL----G---L----AP---SD------AISRLV---SDGGSDNMLRV
NHYPPR-PEL---QAG---------------KN----Q-LL--TGFGEHTDPQIISVLR-
SN---G-TSGLEIASPRD---------G-------AWASVPP----DADS--FFINVGDV
LQVLTNGRFR-----SVKHRV-VV------N--SE-RPRMSMIFFGGPPPGERLAPLR--
-ELLGD------DGG----R-SR---YREFTW-------KEYKST-GCRGR---------
-------------------------------LAEDR--------------------LCLF
EN--
>LOC_Os01g11150.1

--MVVLAKGEL-
-EQIALP---AAHPP------PA---------------------------DVRAIDLSAT

GPARAA-------------------------EARA-LVAACEEQGFFRVTG--HGV----
PPG--LVRAAEAAAARFF-ALPQPDKEAA-------------AGA----PLGYASK-RIG
-SA--GDLGWIEY------LLL--------------CLAPAAAAAALPCA----------
--ATSPTPPCPL-R--------------------------------ELL-REYSAAVRR
VACGVLELMAE-GL----G---V----GP---AD------ALARLV---AREDSDSILRV
NHYPPR-PDQ--LGGG---------------GG----P-NL--TGFGEHTDPQIISVLR-
SN---G-APGLEIS-LRD---------G-------AWASVPH----DGDGDSFFVNVGDT
LQVLTNGRFR-----SVKHRV-VV------N--SE-KSRVSMVFFGGPPPGERLAPLP--
-ALLG-------DGG----R-SR---YREFTW-------KEYKGS-GCKGR---------
------------------------------LADDR--------------------LCRF
EN--
>KQL04928

--MVVLDKGEL-
-EQIALP---AAQPP-----------VA----------------------DVRSVDLSAA
A--GPAREA----------------------AARA-LVAACEEHGFFRVTG--HGV----
PVE--LVRSAEAAAAGFF-ALPQGVKEE--------------EAP----TLGYGSK-QIG
-GN--GDLGWIEY------LLL-GVTPAG-------AVPVATSASSSTLPCAAAAAAAAA
ASWSTSTPAGPL-R--------------------------------DLL-DEYTVAVRR
MACAVLELMEE-GL----G---L----GG---GG------ALARLV---TNEDSDCVLRV
NHYPPR-PAAPELAAG---------------AP----P-NL--TGFGEHTDPQIISVLR-
SN---G-TSGLEIA-LRG---------G-------AWASVPP----DGDA--FFVNVGDT
LQVLTNGRFR-----SVRHRV-VV------N--SE-RSRVSMIFFGGPPPGERLAPLP--
-QLLG-------DGG----R-SR---YLEFTW-------REFKTS-GCRGR---------
------------------------------LAEDR--------------------LSRF
ENN---
>Zm00001d008909_P002

--MVVLAKGEL-
-EQIALP---AAEPP---------PA-----------------------DVRSVDLSAP
A--GPAREA----------------------AARA-LVAACEEHGFFRVTG--HGV----
PAR--LVRAAEAAAAGFF-ARPQGEKEEE-------------GP-----TLGYGSK-RIG
-GN--GDLGWVEY------LLL-GGTPAA---------APSSALLPCAAA----------
--PSPSAPAGPL-R--------------------------------DLL-DEYTVAVRR
MACAVLELMAE-GL----G---I----AGGAGDA------VLARLV---ARADSDCMLRV
NHYPPR-PAL---------------------N-----P-SL--TGFGEHTDPQIISVLR-
AN---G-TSGLEIA-LRD---------G-------AWASVPP----DGDA--FFVNVGDT
LQVLTNGRFR-----SVRHRV-VV------N--SE-KSRVSMVFFGGPPPGERLGPLP--
-QLLG-------DGG----R-SR---YRDFTW-------SEFKTS-GCRTR---------
------------------------------LAEDR--------------------LSRF
EKK---
>Zm00001d039394_P001

--MVVLAKGEL-

-EQIALP---AAAPP-----------RA----------------------DVRSVDLSAP
A--GPAREA----------------------AARA-LVAACEEHGFFRVTG--HGV----
PAE--LVRAAEAAAAGFF-ARPQGEKDG--------------EAP----TLGYGSK-RIG
-GN--GDLGWVEY------LLL-GVNPAV----------PAASASSSTLP----------
-----------L-R---------------------------------GLL-DEYTVAVRR
MACAVLELMAE-GL----G---I----AG---AGDGDGDTVLARLV---ARADSDCMLRV
NHYPPR-PAL---------------------NY----P-CL--TGFGEHTDPQIISVLR-
AN---G-TSGLEVA-LRD---------G-------AWASVPP----DGDA--FFVNVGDT
LQVLTNGRFR-----SVRHRV-VV------N--SE-KSRVSMIFFGGPPPDERLAPLP--
-QLLGD------DGG----R-SR---YRDFTW-------SEFKTS-GCRTR---------
------------------------------LAEDR---------------------LSRF
EKQ---
>EES00081

--MVVLAKGEL-
-EQIALP---AAEPP------PA---------------------------DVRSVDLSAP
A--GPAREA----------------------AARA-LVAACEEHGFFRVTG--HGV----
PPE--LVRAAEAAAAGFF-AQPQDEKDEE-------------AP-----TLGYGSK-RIG
-GN--GDLGWVEY------LLL-GVTPAG-------AAVPAASASSSTLPCAAAAAAATA
SSPSAPAGSAGLLR---------------------------------DLL-DEYTVAVRR
MACAVLELMAE-GL----G---IAGGAGD---GD------VLARLV---TRADSDCMLRV
NHYPPR-PAL---------------------NP----C-SL--TGFGEHTDPQIISVLR-
AN---G-TSGLEIA-LRD---------G-------AWASVPP----DGDG--FFVNVGDT
LQVLTNGRFR-----SVRHRV-VV------N--SE-KSRVSMVFFGGPPPGERLAPLP--
-QLLG-------DGG------RS-------------------------------------

>Zm00001d037724_P002

--MVVLTKGEL-
-EQIALPAVQRASPP------PPA--------------------------AVPEVDLAAG
D--AAA-------------------------AARA-VAKACEDHGFFKVTG--HGV----
PPH--LLARLEAAAAAFF-ALPQREKDR---------------AAG-C--PFGYASK-RIG
-AN--GDLGWVEY------LLL-AVTAAG--------------AAAAPGS----------
--ACEGAEPSCF-R---------------------------------AVL-DEYVAAVRR
MTCTVLQLMAQ-GL----G---L----DD---RD------VFSRLV---LDRDSDSMLRV
NHYPPA-AET---------------------------R-RL--TGFGEHTDPQIISVLR-
SN---D-ASGLEIT-LRD---------G-------TWVSVPS----DTES--FFVNVGDA
LQVLTNGRFR-----SVRHRV-MV------S--SA-RPRVSVIFFGGPPPRERLAPLP--
-GLVDR------EGG----R-RR---YREFTW-------REYKNS-AYRTK---------
------------------------------LADNR---------------------LCSF
ETMATS--
>KXG21531

--MVVLTKGEL-
-EQITLPAVQRAAPP-----------LA----------------------FVPEVDLSAA
A--AS----VAARSA----------------AARA-VAKACEDHGFFKVTG--HGV----
PAP--LLARLEAAAAAFF-ALPQRDKDKAAAAAVG-------GSP----PFGYASK-RIG
-GN--GDLGWVEY------LLL-GVTPAG-----AAAAPSALSAAPVSSA----------
--SEGAAPSCCF-R---------------------------------DVL-DEYIAAVRR
MTCTVLELMAQ-GL----G---L----DDD--TA------VFSRLV---LDRDSDSMLRV
NHYPPR-PETAAAAGE---------------PAEVRRP-RL--TGFGEHTDPQIISVLR-
SN---D-TAGLEIS-LRD---------G-------SWVSVPS----DTQS--FFVNVGDA
LQVLTNGRFR-----SVRHRV-MV------S--SG-RPRVSVIFFGGPPPRERLAPLP--
-GLVDR------EGG----R-RR---YREFTW-------REYKTS-AYRTK---------
-------------------------------LADNR--------------------LCYF
ETTTAAAAATS---
>HORVU1Hr1G029520.3

--MVVLAKGEL-
-EQIALPAVHKTAPP-----------LA----------------------DLPEVDLSVA
R--SG----SEGRAA----------------AARA-VAAACEEHGFFKVTG--HGV----
PAE--LLARVENAAAAFF-ALSQRDKEAAMSAA---------TPG-S--PFGYASK-RIG
-NN--GDLGWVEY------LLL-GVTAAG---------AEAGAPLSVPEE----------
--VVPSASLCSF-R---------------------------------DLL-NEYTVAVRR
MTCQLLELMAE-GL----G---M----ED---RD------AFTRLV---LHKESDSMLRV
NHYPPR-PELKQHHGG-----------------------RV--TGFGEHTDPQIISVLR-
SN---A-TSGLEIA-LRD---------G-------DWVSVPP----DQTS--FFVNVGDA
LQVLTNGRFR-----SVRHRV-MV------N--NI-QPRVSVIFFGGPPPRETLAPLP--
-QLVG-------EGG----R-SR---YKEFTW-------CEYKAS-AYGTK---------
-------------------------------LAANR--------------------LCHF
ETTN--
>KQK07053

--MVVLAQGEL-
-EQIALPAVQKAAPP-----------LA----------------------DVPEIDLAAA
G--AGGS--AAGRAA----------------AAKA-VAAACEEHGFFKVTG--HGV----
ASG--LLARVEAAAADFF-ALPQREKEAAMAMAMAPAAA---AAGSS--PFGYASK-RIG
-SN--GDLGWVEY------LLL------------------GVTAAGAPLP----------
--VPSSASLCAF-R---------------------------------ELL-DEYTVAVRR
MTCEVLELMAE-AL----G---M----E----KD------EFTRLV---LEEDSDSMLRV
NHYPPA-PRPELKQQLQHGGN----------------G-RV--TGFGEHTDPQIISVLR-
SN---A-TSGLEIA-LRD---------G-------TWVSVPP----DHTS--FFVNVGDA
LQVLTNGRFR-----SVRHRV-MV------N--SV-RSRVSVIFFGGPAPGKTLAPLP--
-RMVG-------EGG----S-SR---YREFTW-------REYKAS-AYRTK---------
-------------------------------LAENR--------------------LCHF
ETTS--
>KQL07047

```
------------------------------------------------------------
-----MHLLQQPHYTHAHTTSTPISPVLGVPALFRAVLRPPRVPYRPETAMVVLANPPVV
DQ---IP---LLRSP------GPRDSFA----------------------AVPVVDLSGP
G------------------------------AARA-IVDACERFGFFKVVN--HGV----
AAA--TMDRAETEAIRFF-AQAQADKDRA-------------GPA-Y--PFGYGSK-RIG
-LN--GDMGWLEY------LLL--------------AVDSASLSDACSVP----------
-------SNAAF-R---------------------------------AAL-NEYIAAVRK
VAVRVLEAMAE-GL----G---I----AP---LD------ALSAMV---TEQGSDQVFRV
NHYPPC-PALQGLG-----------------------C-SA--TGFGEHTDPQLVSVLR-
SN---G-TSGLQIA-LRD---------G------AQWVSVPS----DRDA--FFVNVGDS
LQVLTNGRFK-----SVKHRV-VT------N--SL-KSRVSFIYFGGPPLAQRIEPLP--
-ELLG-------EGE----E-SL---YKEFTW-------GEYKKA-AYKTR---------
-------------------------------LGDNR--------------------LAQF
EKK-----------------------------------------------------
>Zm00001d012712_P001
------------------------------------------------------------
-------------------------------------------------MVVVLANPPVV
DQ---IP---LLRSP------GPRDSFS----------------------CVPVVDLSGP
G------------------------------AARA-IVDACERFGFFKVVN--HGV----
PAA--TMDVAESEAVGFF-AQAQADKDRA-------------GPR-ASYPFGYGSK-RIG
-LN--GDMGWLEY------LLL--------------AVDSASLSDACPVP----------
-------SGAAF-R---------------------------------SAL-NEYVAAVRD
VAARVLEAMAE-GL----G---I----AD---AA------ALSSMV---TGAGSDQVFRV
NHYPPC-PALQGLGCT---------------------A-TA--TGFGEHTDPQIISVLR-
SN---G-TSGLQVA-LRD---------A---AQAQQWVSVPS----DRDA--FFVNVGDS
LQVLTNGRFE-----SVKHRV-VT------N--SL-KSRVSFIYFAGPALEQRIAPLA--
-QLLA-------EGE----E-SL---YREFTW-------GEYKTA-AYKTR---------
-------------------------------LGDNR--------------------LAQF
QRCSI---------------------------------------------------
>Zm00001d043411_P001
------------------------------------------------------------
--------------------------------------------------MVVLAKPPVV
DQ---IP---LLRSP------GPRDSFS----------------------GVPVVDLSSH
G------------------------------AARA-IVDACERFGFFKVVN--HGV----
AAA--TMDRAESEAVRFF-AQAQADKDRA-------------GPA-Y--PFGYGSK-RIG
-LN--GDMGWLEY------LLL--------------AVDAASLSDACPVP----------
-------SSAAF-R---------------------------------SAL-NEYVAAVRK
VAARVLEAMAE-GL----G---I----AD---AD------ALSSMV---SGAGSDQVFRV
NHYPPC-PALQGLG-----------------------C-ST--TGFGEHTDPQIISVLR-
SN---G-TSGLQIA-LRD---------G------AQWVSVPS----DRDA--FFVNVGDS
LQVLTNGRFR-----SVKHRV-VT------N--SL-KSRVSFIYFAGPPLGQRIAPLP--
-QVLA-------EGE----E-SL---YKEFTW-------GEYKKA-AYKTR---------
-------------------------------LGDNR--------------------LAQF
EKRSNI--------------------------------------------------
```

>EES01498

--MVVLANPPVV
DQ---IP---LLRSP------GPRDTFS----------------------GVPVVDLSSP
A------------------------------AARA-IVDACERFGFFKVVN--HGV----
PAA--TMGRAESEAVRFF-AQAQADKDRA-------------GPA-Y--PFGYGSK-RIG
-LN--GDMGWLEY------LLL--------------AVDSASLSDACPVP----------
-------STAAF-R---------------------------------SAL-NEYVAAVRK
VAVRVLEAMAE-GL----G---I----AD---AA------ALSSMV---TGAGGDQVFRV
NHYPPC-PALQGLG-----------------------C-SA--TGFGEHTDPQLISVLR-
SN---G-TSGLQIA-LRD---------G------AQWVSVPS----DRDA--FFVNVGDS
LQVLTNGRFK-----SVKHRV-VT------N--SL-KSRVSFIYFAGPALEQRIVPLP--
-ELLA-------EGE----E-SL---YKEFTW-------GEYKKA-AYKTR---------
-------------------------------LGDNR--------------------LAQF
EKRSI---
>LOC_Os01g55240.1

--MVVLAGPPAV
DH---IP---LLRSP------DPGDVFS----------------------GVPVVDLGSP
G------------------------------AARA-VVDACERYGFFKVVN--HGV----
ATD--TMDKAESEAVRFF-SQTQPDKDRS-------------GPA-Y--PFGYGSK-RIG
-FN--GDMGWLEY------LLL--------------ALDDASLADACTVP----------
--------SCAVFR---------------------------------AAL-NEYISGVRK
VAVRVMEAMSE-GL----G---I----AQ---AD------ALSALV---TAEGSDQVFRV
NHYPPC-RALQGLG-----------------------C-SV--TGFGEHTDPQLVSVLR-
SN---G-TSGLQIA-LRD---------G-------QWVSVPS----DRDS--FFVNVGDS
LQVLTNGRFK-----SVKHRV-VA------N--SL-KSRVSFIYFGGPPLAQRIAPLP--
-QLLG-------EGE----Q-SL---YKEFTW-------DEYKKA-AYKSR---------
-------------------------------LGDNR--------------------LAQF
EKK---
>KQK09810

--MVVLASPPAA
DH---IP---LLRSP------DPGDYFS----------------------GMPVIDLCSP
G------------------------------APRA-IADACERFGFFKLVN--HGV----
ATD--AMDRLESEAVTFF-SQPQADKDRS-------------GPA-Y--PFGYGSK-RIG
-LN--GDMGWLEY------LLL---------------AVDDSASLSGAVP----------
-------SGSAF-R---------------------------------RAL-NEYIGAVRK
VAVRVMEAMAE-GL----G---I----AP---LD------ALSKMV---TAAGSDQVFRV
NHYPPC-AALQGLG-----------------------C-SA--TGFGEHTDPQLVSVLR-
SN---G-TSGLQIA--LQ-------DGG-------QWVSVPS----DRDA--LFVNVGDS
LQVLTNGRFK-----SVKHRV-VA------N--SL-KSRVSLIYFGGPPLTQRIAPLP--
-QLLG-------EGE----Q-SL---YTEFTW-------SEYKKA-AYKSR---------
-------------------------------LGDNR--------------------LAQF

QK--
>HORVU3Hr1G072810.1

--MVVLAGTPAV
DH---IP---LLRSP------DPGDNFS----------------------GMPVVDLSRP
G------------------------------APRA-IADACERFGFFKLVN--HGV----
ALD--AMDRLESEAVRFF-SLPQADKDRS-------------GPA-Y--PFGYGSK-RIG
-LN--GDMGWLEY------LLL--------------------AVDSASLP----------
--AASAVPSCALFR---------------------------------AAL-NEYIAAVRK
VAVRVMEAMAE-GL----G---I----AP---AD------ALSAMV---AAEGSDQVFRV
NHYPPC-HALQGLG-----------------------C-SA--TGFGEHTDPQLISVLR-
SN---G-TSGLQIA-LQS---------G-------HWVSVPS----DRDA--FFVNVGDS
LQVLTNGRFK-----SVKHRV-VA------N--SL-KSRVSMIYFGGPALTQRIAPLP--
-QLLR-------EGE----Q-SL---YKDFTW-------GEYKKA-AYNSR---------
-------------------------------LGDNR--------------------LAHF
HR--
>LOC_Os05g43880.1

--MVVLAKPAAL
EQIS------LVRSP------SVEDNFG---------------------AGLPVVDLAAD
G------------------------------AAGE-VVRACERFGFFKVVS--HGV----
GEG--VVGRLEAEAVRFF-ASPQAAKDAH-------------GPA-S--PFGYGSK-RIG
-RN--GDMGWLEY------LLL--------------------AIDGASLS----------
--RSSPAPSSSL-R---------------------------------DAA-NKYVGAMRG
MARTVLEMVAE-GL----G---V----AP---RG------ALADMV-VGDGAASDQILRL
NHYPPCPPLLQNLMPN---------------------C-SP--TGFGEHTDPQLISILH-
SN---S-TSGLQVA--LHHDAD-----A----GDHQWVTVPP----DPAS--FLVIVGDS
LQVMTNGRMR-----SVRHRV-VA------N--KL-KSRVSMIYFGGPPLEQRIAPLR--
-QLLVAGVGNGEEEE----Q-SR---YEEFTW-------GEYKKA-AYLSR---------
-------------------------------LSDNR--------------------LAPF
HRQPPPVANPLA--
>Zm00001d038695_P001

--MVVLAKPPAL
DQISLL----RSPQP------GDASSFP----------------------GVPAVDLSSP
G------------------------------AALA-VVDACERFGFFKVVN--HGV----
PAG--VVDRLEAEAVRFF-ASPQAAKDACGC-----------GPA-S--PLGYGNR-RIG
-RN--GDMGWLEY------LLL-------------ALDGNASVSKASPVP----------
--SSSLRSGPAC-R---------------------------------DAV-NQYVASVRG
LATSVLEAVAE-GL----G---V----AP---RD------ALSGMV---ADAASDQVFRI
NHYPAC-PLLQRLPDS---------------------C-GV--TGFGEHTDPQLVSVLR-
SN---G-TPGLQLA--LH---------G----DDGRWVPVPP----DRDA--FFVIVGDS
LQVLTNGRLK-----SVRHRV-VA------N--SL-KPRVSMIYFAGPAPAQRIAPLP--
-QLLG-------HGK----Q-SL---YRDFTW-------GDYKKA-AYRSR---------

-------------------------------LGDNR--------------------LDPF
RI--
>EES19801

--MVVLAKPPPA
LDQISLL---RCPQP------GDAASFF----------------------GVPAVDLSSP
G------------------------------AALA-VVDACERFGFFKVVN--HGV----
PTG--VVDRLEAEAVRFF-ASPQADKDAC-------------GPA-N--PLGYGNK-RIG
-RN--GDMGWLEY------LLL-------------------ALDGASSVS----------
--KASPVPSSSL-R---------------------------------DAV-NQYVAAVRG
LATSVLEAVAE-GL----G---V----AP---RD------ALSGMV---TDAASDQVFRI
NHYPAC-PLLQRLPDS---------------------C-GV--TGFGEHTDPQLVSVLR-
SN---G-TAGLQVA--LH--DD-----G----G--RWVPVPP----DRDA--FFVIVGDS
LQVLTNGRLK-----SVRHRV-VA------N--SL-KPRVSMIYFAGPAPAQRIAPLP--
-QVLG-------HGE----Q-SL---YRDFTW-------GDYKKA-AYRSR---------
-------------------------------LGDNR--------------------LDPF
RIQ---
>KQK05406

--MVVLAKPAAL
EQISLLR------TP------EPWESFA----------------------GVPAVDLSAP
G------------------------------AADD-VVRACERFGFFSVVN--HGV----
ARG--VVERLEHEAALFF-SWPQADKDASAS-----------GPA-D--PFGYGSK-RIG
-RN--GDMGWLEY------LLL--------------------AIDRESLS----------
--KASPAPSSSL-R---------------------------------DAI-NEYVGAMRG
LARTVLEMVAE-GL----G---V----SP---RG------ALADMV---TGEASDQVFRL
NHYPPC-PLLQGLPPN---------------------C-SV--TGFGEHTDPQLVSILH-
SN---A-TAGLQIA--LH--GD-----G----DAQKWVSVPP----NRDA--FFVNVGDS
LQVLTNGRLK-----SVRHRV-VA------G--SGRKSRVSMIYFGGPPVAQRIAPLP--
-QLLQ-------AEG----QLPI---YREFTW-------GEYKKA-AYRSR---------
-------------------------------LGDNR--------------------LAPF
QMTLVELQHAHADAAGLHHRS-----------------------------------
>HORVU1Hr1G076730.2

--------APPHLELLSPVLHCCDSSSLYGVSGSGTDRFALPLLCLAQLSSVSFLAMVVL
AKPAALEQIALMRTP------EPWESFS----------------------GVPAVDLSSP
G------------------------------AAAD-VVRACERFGFFSVVN--HGV----
PAG--VVDRLEAEAVRFF-ASTQAEKDAS-------------GPA-D--PFGYGSK-RIG
-RN--GDMGWVEY------LLL--------------------AIDRDTLS----------
--KASPAPSSAL-R---------------------------------EAI-NAYVSAMRG
LARTVLEMVAE-GL----G---V----SP---RG------ALADMV---TGEASDQVFRV
NHYPPC-PLLQGLPPN---------------------C-SV--TGFGEHTDPQLVSILH-
SN---G-TAGLQVA-LHD---------G-------RWVSVPP----NRDA--FFVNVGDS
LQVLTNGRLR-----SVRHRV-VA------G--NGLKSRVSMIYFAGPPLAQRIAPLQ--

-QLLA-------GTQ----SLPL---YRDFTW-------GEYKKA-AYRSR---------
------------------------------LGDNR--------------------LAPF
ETPLVAMPHAAHHRS--
>HORVU1Hr1G086710.2

----------------------------MTTTANVPLLLTSLLLLLCFTYISVLCEIVL
LAMVGVTVPISVDTI------PLVKCAH------------------AAAAAVPSVDLSAP
D-----------------------------AGAA-VAAACRRVGFFRATN--HGV----
PAA--LTDALEARAEAFF-ALPHEDKLEA-------------SAR----PFGYGSK-SIG
-CN--GDVGWLEY------ILL--------------SVGSGSVAAACLPP----------
--SL---------R--------------------------------AAL-EEYTDAVRE
VGARVLELMAD-GL----G---V--AEEH---RG------VLRQMA---EADGADEMVRV
NHYPPC-PCPLAAGQR-----------------------GV--TGFGEHTDPQIISVLR-
SN---R-TGGLQIA-LPD---------G-------RWVPVAP----DPDS--LFVNVGDS
LQVLTNGRFR-----SVKHRV-VA------PAEGQ-QPRLSVIYFGGPAPTQRIAPLP--
-ELMR-------EGE----Q-SL---YRDFTW-------AEYKKA-AYKSR---------
------------------------------LGDHR--------------------LGPF
ELPAAEEPTSSDKHCGSNAVQLPAPAPLHVARVH---------------------
>HORVU1Hr1G086810.2

--TVAITVPISV
DTIPLLK--------------CVNAATA----------------------AVPSVDLSAP
G-----------------------------AAAA-VADACRAVGFFRATN--HGV----
PAA--LTDALEERAASFF-ALPHKDKMDA-------------SAR----PFGYGSK-NIG
-CN--GDVGWLEY------ILL------------------SVRSGSVAAA----------
--SLPPSLRAAL---------------------------------------EEYTDAVRE
VGARVLELMAD-GL----G---V--AEEH---RG------VLRRMVAPDDAGGADEMVRV
NHYPPC-PSSLAAGQR-----------------------GV--TGFGEHTDPQIISVLR-
SN---R-TAGFQIM-LPD---------G-------RWVPVAP----DTDS--LFVNVGDS
LQVLTNGRFR-----SVKHRV-VA------PTEGQ-QPRLSVIYFGGPAPTQRIAPLP--
-ELMR-------EGE----Q-SL---YRDFTW-------AEYKKA-AYKSR---------
------------------------------LGDHR--------------------LGPF
ELPAAATAQESTTKADHYCSSNAAVQAPAAAPHVARVH------------------
>KQK04908

---MAAITMTALPSSV
DHQVVIP---LLKCP----IAAAAAAAM----------------------IPTVDLSSP
G-----------------------------AARA-VAEACRGVGFFRATN--HGV----
PSS--LAATLEADAMAFF-ALPDKDKQSTTTTTTPAP-----GPGSARPSLGYGSR-RIG
-SN--GDVGWLEY------LIL-------------------SLGSNSVLP---------
---------ASL-R--------------------------------LAL-HEYTRAVRE
LSGRVLELMAE-GL----G---I----VGETERG------VLRRMV----AGSEEELVRV
NHYPPP-TTERDEDDD---------------GC----V-GI--TGFGEHTDPQLISLLR-
SN---R-TAGYQIL-LQE---------A----DEARWVNVAP----DPDS--FFVNVGDT

LQVLTNGRFR-----SVKHRVLVA------PERGGKASRLSVIYFGGPAPAQRIAPLP--
-ELMR-------EGE----R-SL---YRDFTW-------GEYKAA-AYKTR---------
-------------------------------LGDHR--------------------LGPF
QVQLLPPAAAKA--
>KQK04914

--MVAIKAPSSI
DHANPLT-----KSP------KAAAAAA---------------------AAIPTVDLSSP
G------------------------------AARA-VADACRGVGFFRATN--HGI----
PSS--LAAALEARAMAFF-ALPHEDKVDATI-----------AAA-ARRPFGYGSR-SIG
-SN--GDVGWLEY------LLL----------------SLGSNSNSSSIP----------
--GASSLPPSLR-----------------------------------AAL-EEYTGAVRN
ASGKVLELMAE-GL----G---M----QE---RC------ALRRMV----DGSEEELVRV
NHYPPT-KEEDCV------------------------A-GM--TGFGEHTDPQIISLLR-
SN---R-TAGLQIK--LQ---------G----PDAPWVNVAP----DPDS--LFVNVGDC
LQVLTNGRFR-----SVKHRV-VA------PEGAQ-ASRLSVIYFGGPAPAQRIAPLP--
-ELMR-------EGE----Q-SL---YRDFTW-------GEYKAA-AYKTR---------
-------------------------------LGDNR--------------------LGPY
ELRNVADTNPTALITASKEPTAADHCCSNSSSSSAACVVVQPPHVAQVH-------
>LOC_Os05g48700.1

--MVAITAPSSI
EH---IP---LVRCP------KGANAGP---QA-----------------VIPCIDLSAP
G------------------------------AAAA-VADACRTLGFFKATN--HGV----
PAG--LADALESSAMAFF-ALPHQEKLDMS------------GPA-R--PLGYGSK-SIG
-SN--GDVGWLEY------LLL---------------SAGAASSGGAALP----------
---------AAL-R---------------------------------AAV-EAYTGAVRG
VGCRVMELMAE-GL----G---LGASEEG---RC------VLRRMV--VGCEGSDEMLRV
NHYPPC-LLPPGRDRDE--------------------C-GV--TGFGEHTDPQIISVLR-
SN---C-TAGLQIL--LR---GDYSSPA-------RWVPVPP----DPDS--FFVNVGDS
LQVLTNGRFR-----SVKHRV-LA------P--EGEESRLSVIYFGGPAASQRIAPLE--
-QVMR-------EGE----Q-SL---YREFTW-------GEYKKA-AYKTR---------
-------------------------------LGDNR--------------------LGPY
ELQHAAANDEAATKK---
>KQL14341

--MVAITAPSSI
DQ---IP---LMRCP------KANAGQA--------------------AAAIPCVDLSAP
G------------------------------AAAA-VADACRSVGFFRATN--HGV----
PAS--VADALEAGAMAFF-ALPAQDKVDMSG-----------AAR----PLGYGSK-SIG
-SN--GDVGWLEY------LLL------------------SVSANSVKIS----------
--SLPPSLRAAL---------------------------------------EEYTAAVRE
VGGRVLELIAE-GL----G---V----D----RA------LLRSMV--VGREGGDELVRV
NHYPPC-PLRAPGD-----------------------C-GV--TGFGEHTDPQIISVLR-

SN---C-TAGLQIK-LRD---------G-------RWVPVPP----DPES--FFVNVGDS
LQVLTNGRFR-----SVKHRV-VA------P--EGSHSRLSVIYFGGPAPSQRIAPLP--
-QVMR-------DGE----Q-SL---YREFTW-------GEYKRA-AYETR---------
-------------------------------LGDHR--------------------LGSF
ELRAASEPAGGADPQPHCSNSSTCMPPQQQQQVAQVY-------------------
>Zm00001d038996_P001

--MVAITAPSSI
EQIPLMR---CPRAN------AVQQAGA----------------------AVPCVDLSAP
G------------------------------AGAA-VADACRSVGFFRATN--HGV----
PAR--VADALEARAMAFF-ALPAQEKLDMSG-----------AAR----PLGYGSK-SIG
-AN--GDVGWLEY------LLL------------------SVSANTVKIS----------
--SLPPSLRAAL---------------------------------------EEYTAALRE
VCGRVLELIAE-GL----G---V----E----RS------LLRAMV--VGREGSDEVVRV
NHYPPC-PLLPPVD-----------------------C-GV--TGFGEHTDPQIISVLW-
SN---R-TAGLQIK-LRD---------G-------RWVPVPP----TPES--LFVNVGDS
LQVLTNGRLR-----SVKHRV-VA-PAPAPD--GA-RSRLSVIYFGGPAPSQRIAPLP--
-QVMR-------DGE----Q-SL---YREFTW-------AEYKRA-MYKTR---------
-------------------------------LADHR--------------------LGPF
ELRASTNGGNRPGEPAAGSADPAHCSGGSACGMPQPQQVARVH-------------
>EES19940

--MVAITAPSSI
EQ---IP---LVQCP----RANASA-------------------------AIPCVDLSAP
G------------------------------AAAA-VADACRGVGFFRATN--HGV----
PAR--VVEALEARAMAFF-ALPAQEKLDMS------------GAA-R--PMGYGSK-RIG
-SN--GDVGWLEY------LLL------------------SVSANTVKIS----------
--SLPPSLRAAL---------------------------------------EEYTAAVRE
VCGRVLELIAE-GL----G---V----D----RS------LLRAMV--VGREGSDELVRV
NHYPPC-PLLPPVD-----------------------C-GV--TGFGEHTDPQIISVLR-
SN---S-TAGLQIK-LRD---------G-------RWVPVPP----APES--FFVNVGDA
LQVLTNGRFK-----SVKHRV-VA------P--EGAQSRLSVIYFGGPAPSQRIAPLP--
-EVMR-------DGE----Q-SL---YREFTW-------AEYKTA-MYKTR---------
-------------------------------LADHR--------------------LGPF
ELRATNTNSCVPPPPPPSVDPYCNGSGICMPQPPPQQQQVAEVH------------
