# Supplementary figures and images for: Genome-Wide Identification and Evolution Analysis of the Gibberellin Oxidase Gene Family in Six Gramineae Crops
Source: Genes (Basel). 2022 May 12;13(5):863. doi: 10.3390/genes13050863 (PMC9141362; doi:10.3390/genes13050863)

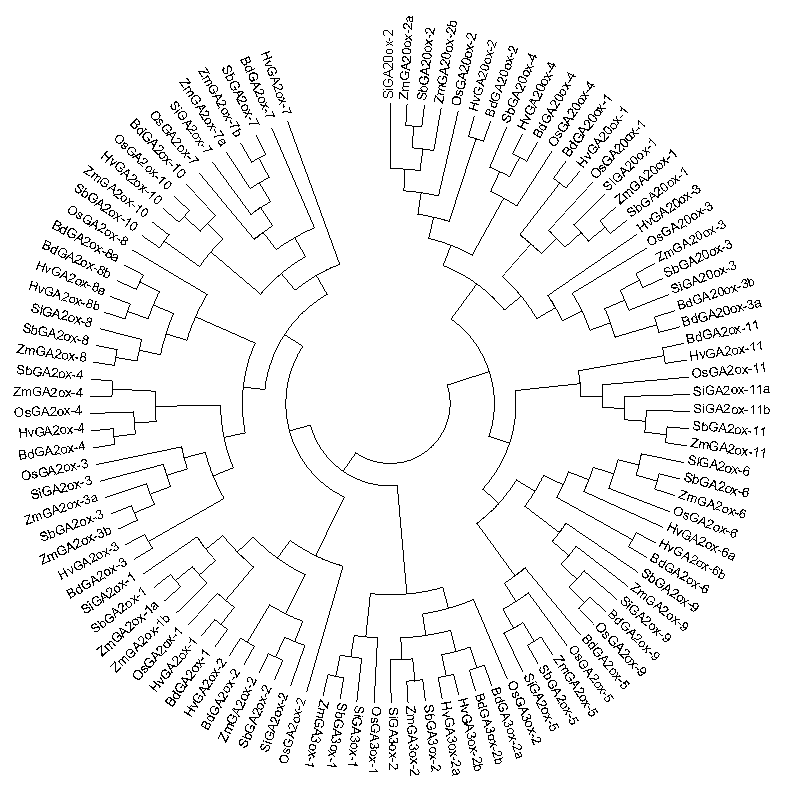

Supplement: Supplementary file 1 [file genes-13-00863-s001.zip › Figure S1.PNG]

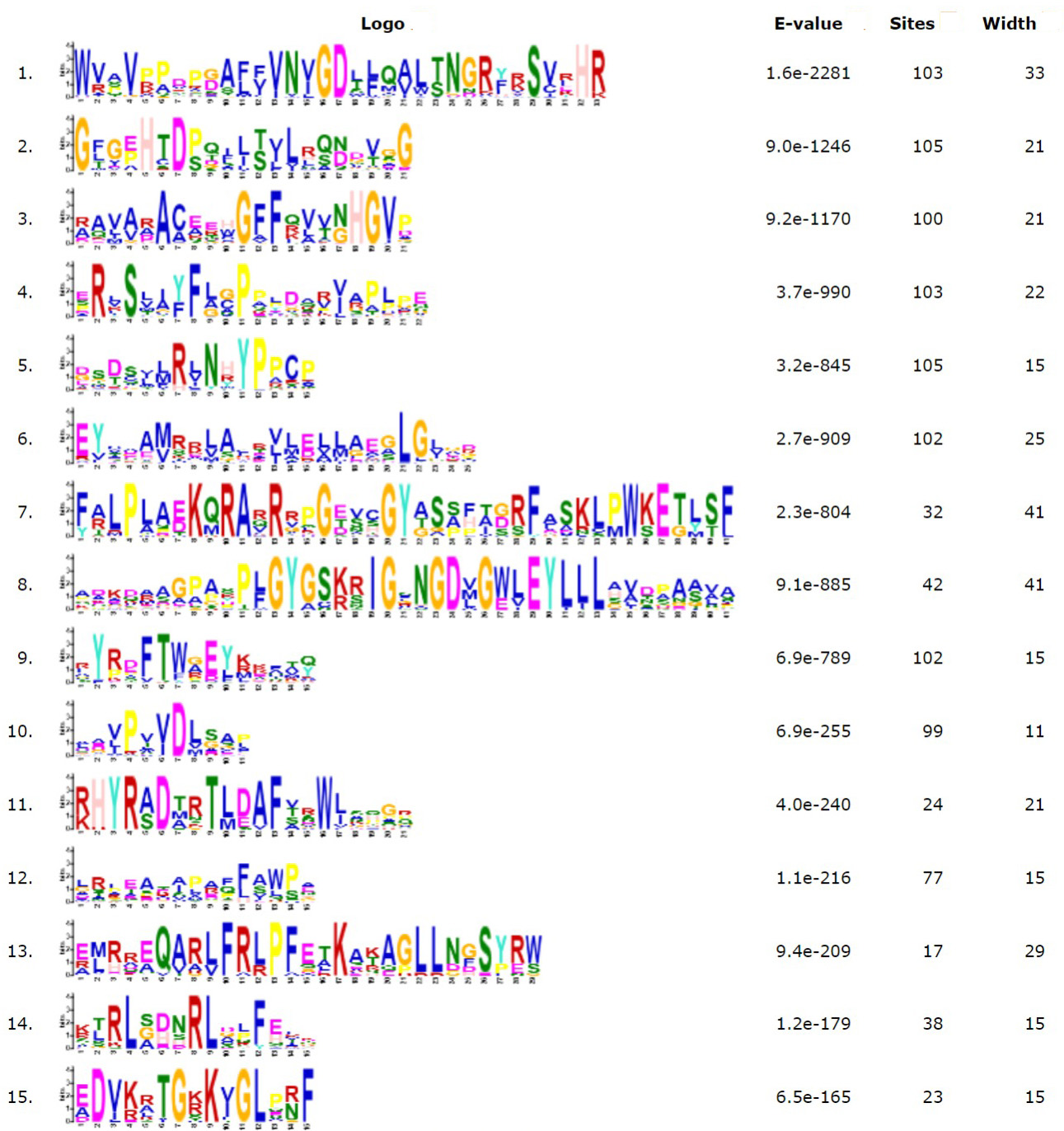

Supplement: Supplementary file 1 [file genes-13-00863-s001.zip › Figure S2.jpg]

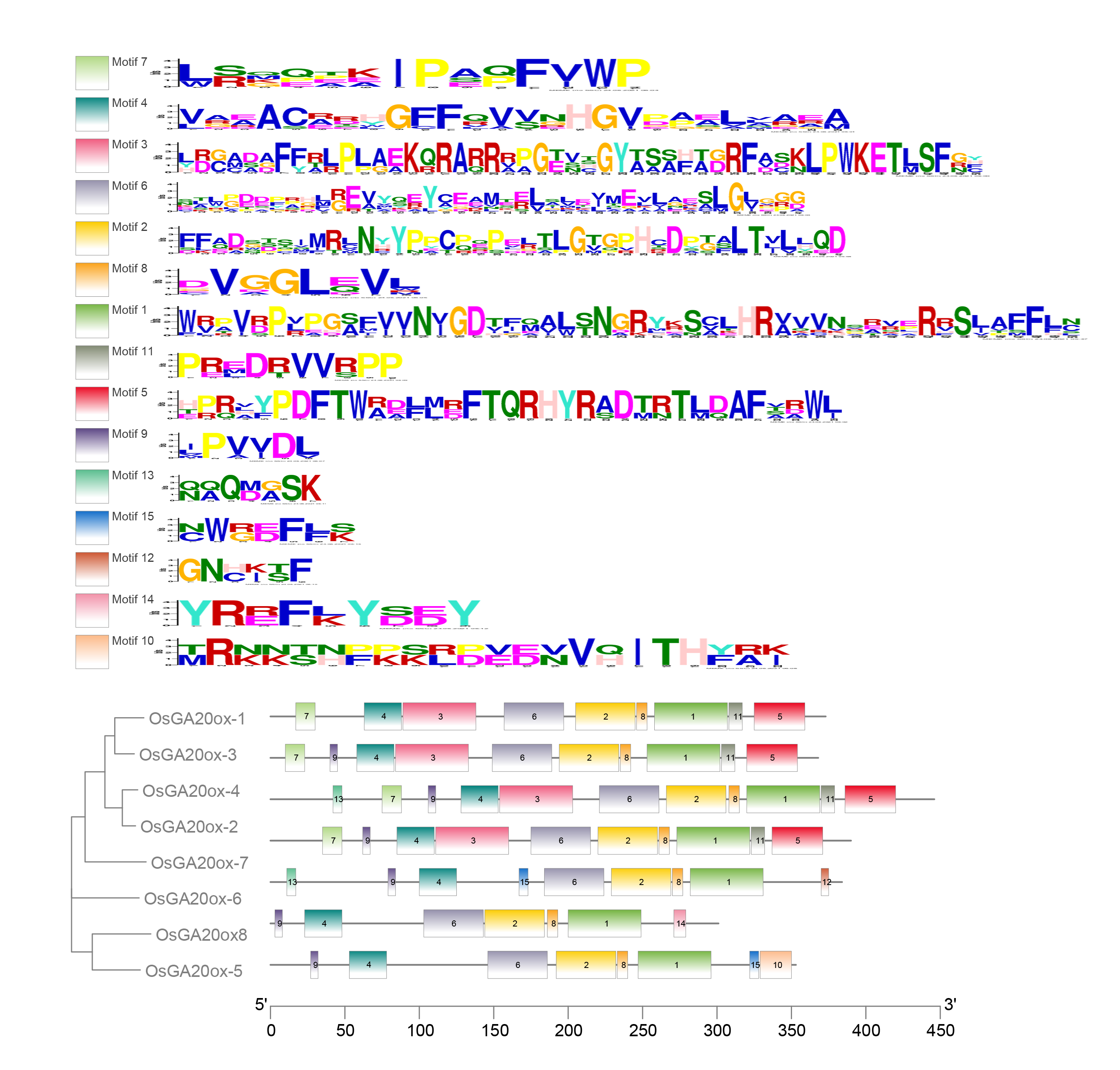

Supplement: Supplementary file 1 [file genes-13-00863-s001.zip › Figure S3.png]

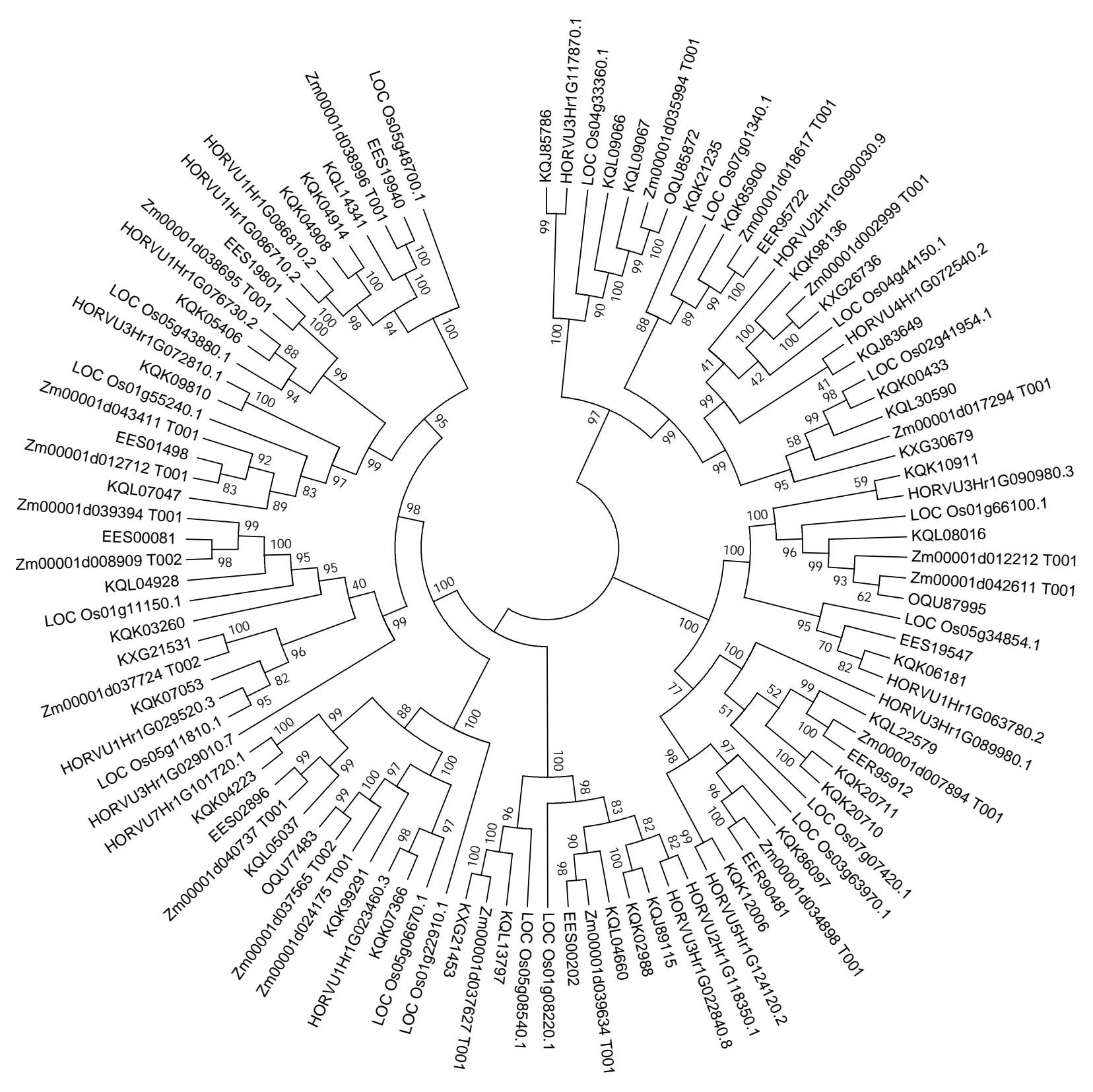

Supplement: Supplementary file 1 [file genes-13-00863-s001.zip › Supplementary file 3.PDF]

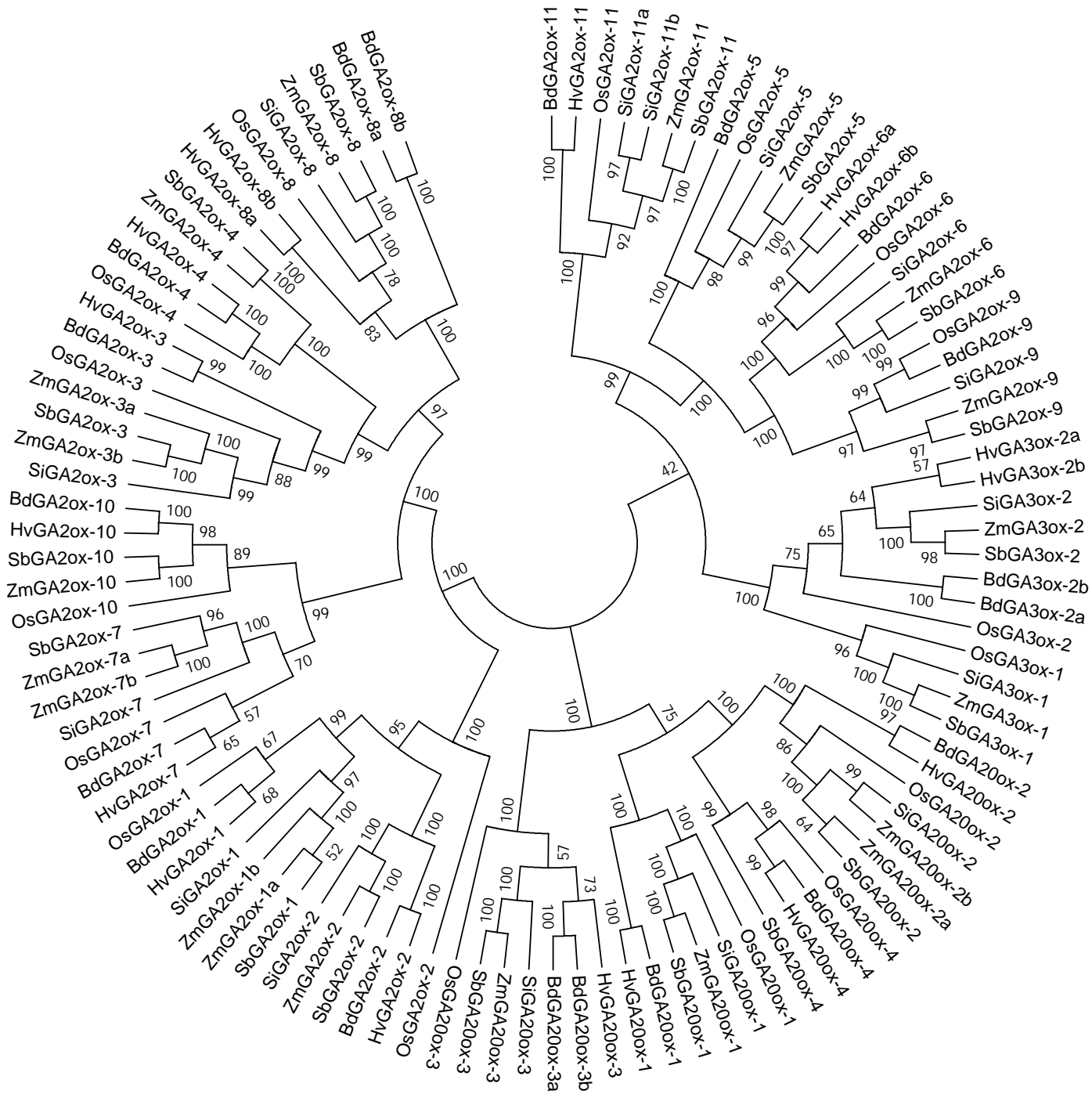

Supplement: Supplementary file 1 [file genes-13-00863-s001.zip › Supplementary file 4.PDF]

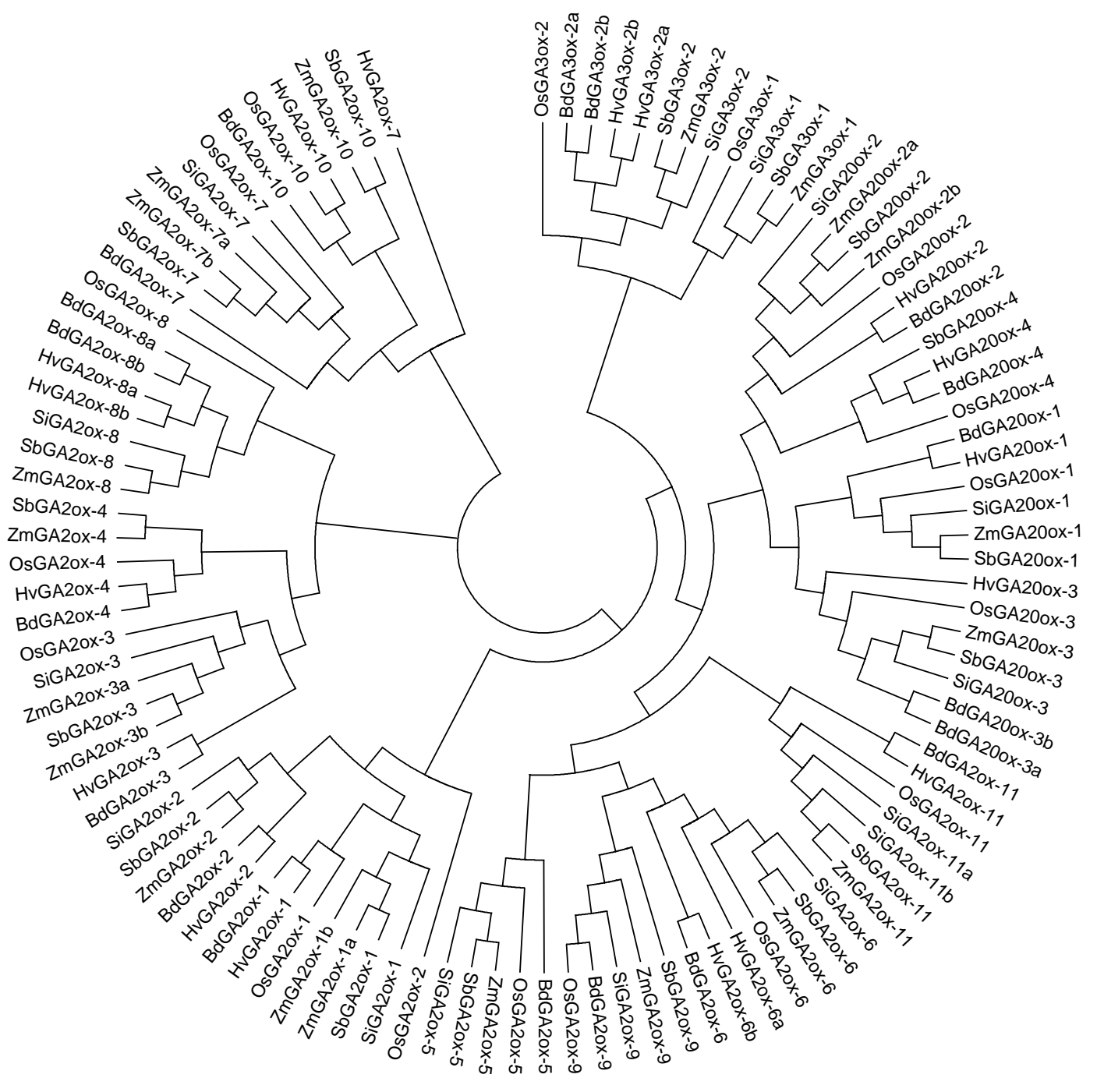

Supplement: Supplementary file 1 [file genes-13-00863-s001.zip › Supplementary file 5.PDF]
